# Antimicrobial Activity and Identification of the Biosynthetic Gene Cluster of X-14952B From *Streptomyces* sp. 135

**DOI:** 10.3389/fmicb.2021.703093

**Published:** 2021-08-02

**Authors:** Na Li, Simin Chen, Zhiqiang Yan, Jinhua Han, Yongquan Ta, Taixun Pu, Yonghong Wang

**Affiliations:** ^1^Research and Development Center of Biorational Pesticides, Key Laboratory of Plant Protection Resources and Pest Management of Ministry of Education, Northwest A&F University, Xianyang, China; ^2^Institute Vegetable, Zhangye Academy of Agricultural Sciences, Zhangye, China; ^3^College of Plant Protection, Northwest A&F University, Xianyang, China

**Keywords:** *Streptomyces*, strain identification, X-14952B, antimicrobial effects, biosynthetic gene clusters

## Abstract

The bacterial genus *Streptomyces* is an important source of antibiotics, and genome mining is a valuable tool to explore the potential of microbial biosynthesis in members of this genus. This study reports an actinomycete strain 135, which was isolated from Qinghai-Tibet Plateau in China and displayed broad antimicrobial activity. The fermentation broth of strain 135 displayed strong antifungal activity (>70%) against *Sclerotinia sclerotiorum*, *Botrytis cinerea*, *Valsa mali*, *Phytophthora capsici*, *Glomerella cingulata*, *Magnaporthe grisea*, *Bipolaris maydis*, *Exserohilum turcicum in vitro*, meanwhile possessed significant preventive and curative efficacy against *S. sclerotiorum*, *Gaeumannomyces graminis*, and *P. capsici* on rape leaves (54.04 and 74.18%), wheat (90.66 and 67.99%), and pepper plants (79.33 and 66.67%). X-14952B showed the greatest antifungal activity against *S. sclerotiorum* and *Fusarium graminearum* which the 50% inhibition concentration (EC_50_) were up to 0.049 and 0.04 μg/mL, respectively. Characterization of strain 135 using a polyphasic approach revealed that the strain displayed typical features of the genus *Streptomyces*. 16S rRNA gene sequencing and phylogenetic analysis demonstrated that the isolate was most closely related to and formed a clade with *Streptomyces huasconensis* HST28^T^ (98.96% 16S rRNA gene sequence similarity). Average nucleotide identity (ANI) and DNA-DNA hybridization (DDH) values in strain 135 and related type strains were both below the threshold of species determination (91.39 and 56.5%, respectively). OrthoANI values between strain 135 and related type strains are under the cutoff of determining species (<95%). The biosynthetic gene cluster (BGC) designated to X-14952B biosynthesis was identified through genome mining and the possible biosynthesis process was deduced.

## Introduction

Actinomycetes have excellent development potential as a microbial resource. Numerous structurally diverse natural products have been isolated from various actinomycetes, and almost two-thirds of natural antibiotics and anticancer agents, pesticides, and antimicrobial agents used in medicine are produced by members of this bacterial group. Actinomycetes play a vital role in bioengineering, medicine, agriculture, and other industries (Aminov, 2017). *Streptomyces*, as the most prominent genus and the most resourceful producer of antibiotics among the actinomycetes, has attracted considerable interest from the beginning of the golden age of natural product-based drug discovery. With the continuous search in discovering new species of *Streptomyces* and the ever-increasing studies of natural product separation, known bioactive compounds have been rediscovered even from phylogeny distinct *Streptomyces*. Therefore, the latest research trends have focused on developing new screening technologies for efficient dereplication and investigating rare samples from extreme and unexplored environments where microorganisms may have unique characteristics compared with those of known species and thus might produce novel bioactive secondary metabolites (Suzuki, 2001). Extreme and unexplored environments include marine environments (Yang et al., 2020), mountains (Wei et al., 2020), deserts (Sivakala et al., 2021), and rainforests (Schneider et al., 2018).

Understanding and analyzing genetic information suitable for producing secondary metabolites is the basis for exploring potential natural products. The development of sequencing technology has led to rapid, accurate, and relatively inexpensive sequencing. Furthermore, the development of computing algorithms and the expansion of secondary metabolite-related databases further facilitate the mining and study of biosynthetic pathways of natural products through the genome by automatically scanning and annotating unique gene clusters of metabolites.

In the process of searching for bioactive secondary metabolites from alpine microorganisms, we isolated an actinomycete species, strain 135, with broad-spectrum antimicrobial activity and selected this strain for further exploration of its specific antimicrobial substances. After large-scale fermentation and purification, the known compound X-14952B was isolated from strain 135. X-14952B is a 20-membered glycosylated macrolide, first reported in 1985 (Omura et al., 1985) and with a similar structure to venturicidin A, B (Brufani et al., 1971), C (Shaaban et al., 2014), 17-hydroxy-venturicidin A (Fourati-Ben Fguira et al., 2005), irumamycin (Omura et al., 1982), 3′-O-decarbamoylirumamycin (Nakagawa et al., 1985), irumanolide (Sadakane et al., 1983), and so on. These compounds have significant antifungal and antitrypanosomal activities, weak antibacterial activities, and low cytotoxicity. Furthermore, these antimicrobial substances can inhibit F_0_F_1_-ATPase and ATP synthesis in both fungi and bacteria (Shaaban et al., 2014).

Although there are some previously published reports on X-14952B, most have been related to the antimicrobial activities of this compound and there are very few studies reporting the synthetic gene cluster of X-14952B and its homologs. One recent study described the biosynthetic gene cluster (BGC) structure of the homologous compound venturicidin A and the possible biosynthesis process of venturicidin A (Li et al., 2021). However, although X-14952B has a similar backbone to venturicidin A, and they are both synthesized by type I polyketide synthases (PKSs), their BGCs are quite different. The module structure of bacterial type I PKSs makes it possible to deduce an unknown compound by bioinformatics analysis of the PKS genes. The limited reports on X-14952B, but plentiful reports on venturicidin suggest that the producer of X-14952B, *Streptomyces* sp. 135, isolated from Qinghai-Tibet Plateau, might be present a potential novel species of the genus *Streptomyces*. Accordingly, this paper first describes the isolation and antimicrobial assays of strain 135, including evaluation on 17 fungi, 2 oomycetes, and 6 bacteria *in vitro* and three fungi *in vivo*. We also evaluated X-14952B derived from strain 135 fermentation broth on 8 plant pathogenic fungi and six bacteria. The 50% inhibition concentration (EC_50_) and minimum inhibitory concentration (MIC) were assayed. The second part is involved with strain identification. Strain 135 was analyzed for cultural, morphological, physiological, biochemical characteristics, whole-genome sequencing and phylogenetic analysis. The third part focuses on the analysis of BGCs related to X-14952B, and the deduction of its unique and specific biosynthesis process.

## Materials and Methods

### Soil Sampling

The sample collection comprised 73 soil samples from 15 different sites within a varied natural environment. Table 1 presents sampling locations and their surrounding conditions. A snake-shaped sampling strategy was chosen, and multiple sampling points were selected in each sampling area. After removing 5 cm of topsoil, soil samples from up to 20-cm depth were collected using a sterilized spatula and placed in sterile, zipped, polyethylene plastic bags. Sample bags were labeled with sampling numbers, data and sampling sites, samplers, and other information.

**TABLE 1:** Source of soil materials collected from Qinghai-Tibet Plateau.

SN	Sampling site	Longitude	Latitude
1	Qinghai Lake	36°31′38.93″–37°0′12.64″	99°49′29.76″–100°45′35.42″
2	Chaka	36°38′29.01″–36°45′2.82″	99°0′53.58″–99°11′58.44″
3	Sichuan-Tibet highway	29°37′55.96″–29°48′23.41″	91°2′40.34″–91°29′10.22″
4	Danggula Mountains	34°12′51.98″–34°12′55.66″	92°25′56.24″–92°27′3.73″
5	Amdo	32°15′15.02″–32°17′54.01″	91°40′30.04″–91°43′32.78″
6	Quxu	29°18′45.53″–29°22′6.41″	90°39′3.08″–90°50′19.97″
7	Lhasa	29°37′45.90″–29°39′23.96″	91°0′48.81″–91°13′12.18″
8	Gangcha	37°15′56.75″–37°19′43.93″	99°49′49.52″–100°23′3.19″
9	Golmud	36°22′39.58″–36°25′19.25″	94°52′30.40″–94°56′49.08″
10	Damxung	30°25′9.41″–30°30′15.72″	91°1′18.82″–91°13′24.62″
11	Cambra	36°6′51.83″–36°6′52.83″	101°49′6.96″–101°49′17.27″
12	Hoh Xil	35°25′59.45″–35°28′33.09″	93°35′10.86″–93°38′37.51″
13	Wudaoliang	35°11′20.19″–35°17′21.37″	93°16′44.21″–93°4′9.41″
14	Naggu	31°23′35.56″–31°31′33.20″	91°54′27.14″–92°4′46.02″
15	Tuotuo river	33°51′18.54″–33°52′31.39″	91°54′17.93″–91°58′18.79″

*SN: site number.*

### Selective Isolation of Actinomycetes

Isolation of actinomycetes was conducted according to the procedure of Suzuki with slight modifications (Suzuki, 2001) to ensure that the range of varieties of genera isolated was as broad as possible and included those that were rarely isolated. Briefly, soil samples were air-dried at 120°C for 60 min, ground, and sieved through 200 mesh sieves. One gram of soil was weighed and soaked with 100 mL solution (0. 1% skimmed milk in 5 mM CHES (2-(Cyclohexylamino) ethanesulfonic acid, pH 9) for 60 min, centrifuged for 20 min at 1,500 rpm, and still set for 10 min. To maximize the isolation of culturable bacterial species, dilutions were plated on the following media supplemented with actinomycin (50 mg/L) and nalidixic acid (50 mg/L) to inhibit growth of fungi and bacteria without affecting the growth of actinobacteria: (1) ISP2 media; (2) ISP4 media; (3) TPA media; (4) HVA media; and (5) HSG media. Compositions of these media are listed in the Supplementary material. All resulting isolates were tentatively identified and classified (aerial/substrate) based on standard morphological criteria like the absence or presence of aerial mycelium, the presence of fragmentation of substrate mycelium, etc. Actinomycetes were isolated and purified in ISP4 medium, and isolates were preserved at 4°C for short-term storage and in glycerol suspensions (20%, v/v) at −20°C for long-term storage.

All strains involved in this experiment were stored in the Shaanxi Research Center of Biopesticide Engineering & Technology culture collection.

### Antimicrobial Assays *in vitro*

Antimicrobial activity of the isolates was tested using a two-stage fermentation process to obtain a cell-free media containing potential metabolites. Briefly, tested actinomycetes were cultured on ISP2 medium plates at 28°C for 7 days, then firstly inoculated into 150 mL ISP4 liquid media using a 250 mL Erlenmeyer flask with shaking (170 rpm) at 28°C for 3 days for the seed culture. In the second stage, 10% seed culture was transferred to 150 mL millet media and left to ferment for 7 days under identical conditions. The fermentation broths were then centrifuged at 8,000 rpm, 4°C for 30 min to make cell-free broth. The ingredients of these media are listed in the Supplementary material

Sixteen plant-pathogenic fungi and oomycetes, including *Alternaria alternata*, *Bipolaris maydis*, *Botrytis cinerea*, *Exserohilum turcicum*, *Fusarium graminearum*, *Fusarium oxysporum*, *Glomerella cingulata*, *Gaeumannomyces graminis*, *Magnaporthe grisea*, *Phytophthora capsici*, *Phytophthora infestans*, *Rhizoctonia solani*, *Sclerotinia sclerotiorum*, *Thanatephorus cucumeris*, *Valsa mali*, and *Verticillium dahliae* were used to test antifungal activity.

The inhibitory effects of metabolites on the mycelial growth of pathogenic fungi and oomycetes were determined by the inhibition mycelial growth rate method as described by Zhang et al. (2019) with a few alterations. Cell-free fermentation broth (5 mL) was mixed well with molten PDA medium (45 mL) at approximately 40 to 50°C and poured evenly into three 90-mm diameter Petri dishes. Added the same amount of sterile water in PDA medium as blank control. *S. sclerotiorum* and *B. cinerea* were used to test all fermentation broth activities of actinomycetes isolated, *A. alternata*, *B. maydis*, *B. cinerea*, *E. turcicum*, *F. graminearum*, *F. oxysporum*, *G. cingulata*, *G. graminis*, *M. grisea*, *P. capsici*, *P. infestans*, *R. solani*, *S. sclerotiorum*, *T. cucumeris*, *V. mali*, and *V. dahliae* were used to test fermentation broth activities of strain 135. A 5 mm mycelial plug of different fungi was placed in the center of each plate then cultured at 25°C in the dark condition until the mycelium reached the edge of the dish on the blank control.

For determination of the antifungal activity of the isolated compound, the mycelial growth rate method was utilized (Zhang et al., 2019). The compound was dissolved in methanol and a stock solution of 20 mg/mL was prepared. The stock solution (150 μL) was diluted with methanol and blended with 15 mL melted PDA agar medium to the required concentration, then immediately poured into 60-mm diameter Petri dishes. Carbendazim (the EC50 was determined) and procymidone (500 mg/L) were used as positive controls and PDA media supplemented with an equal amount of methanol (150 μL) was used as the blank control. After cooling, a mycelial plug with a 5-mm diameter was placed in the center of each plate. Each treatment was repeated three times. The plates were cultured at 25°C in the dark until the mycelium reached the edge of the dish on the blank control. The inhibition rate of mycelium growth was calculated as follows:

$$\mathrm{Inhibition}\mathrm{rate}\left(\%\right)=\frac{\mathrm{Dc}-\mathrm{Dt}}{\mathrm{Dt}-0.5}\times 100$$

where Dc is the mycelial growth diameter of the control, and Dt is the mycelial growth diameter of the treatment. The diameter of the mycelial plug is 0.5 mm.

Seven bacterial species, including *Bacillus subtilis*, *Erwinia carotovora*, *Pseudomonas syringae*, *Ralstonia solanacearum*, *Xanthomonas juglandis*, *Xanthomonas citri*, and *Staphylococcus aureus*, were used to test antibacterial activity.

The agar-diffusion method was adopted to test the antibacterial activities of the actinomycetes isolates (Bonev et al., 2008). Melted NA media (40°C) was supplemented with a volume fraction of 1.5% bacterial suspension, mixed well, and poured into a 90-mm diameter Petri dish as the bacteria-carrier plate. After cooling, 100 μL cell-free broth was added into the hole plugged by a punch (9 mm diameter) and cultured at 25°C for 24 h, measuring the diameter of the inhibition zone.

The broth microdilution method was used to detect the MIC of the isolated compound (Liu et al., 2006). Bacterial suspensions (OD_600_ = 0.8, 200 μL) were added to a 96-well microtiter plate. The positive control, streptomycin (1 mg/mL), and the blank control were separately treated with 0.5% methanol, in other wells added 1 μL stock solution and 199 μL bacterial suspensions. Plates were incubated at 25°C for 48 h then 50 μL iodonitrotetrazolium solution (INT, 1 mg/mL) was added to each well. INT is a bacterial staining material that can stain viable cells red. The minimal inhibitory concentration (MIC) was determined as the lowest concentration of X-14952B, for which the bacterial suspension did not produce a color change (yellow) after addition of INT as mentioned above.

Statistical analyses were computed by SPSS v22.0 (IBM, NY, United States). *P* < 0.05 was considered statistically significant using the least significant difference test to analyze the analysis of variance (ANOVA).

### Antifungal Assays Against *Sclerotinia sclerotiorum*, *Gaeumannomyces graminis*, and *Phytophthora capsici in vivo*

The detach leaf method described by Arfaoui et al. (2018) with *S. sclerotiorum* and pot experiments with *G. graminis* and *P. capsici* were conducted to assess the antifungal activities of strain 135 *in vivo*. Rape (*Brassica napus* L.) (Qingyou-10) was planted in nine individual pots and maintained in a greenhouse at 26°C with a 12 h photoperiod for 30 days. Groups of nine leaves with similar growth were then collected to assay the protective and curative activity. All leaves were washed using sterile water before the test.

For the protective activity assay, one group of leaves was firstly sprayed with the cell-free fermentation broth, carbendazim (800 mg/L) and sterile water were used as positive control and blank control, respectively. A 5 mm mycelial plug of *S. sclerotiorum* was incubated on the surface in the center of each leaf, which was slightly wounded. The petiole was wrapped with degreasing cotton dropped with sterile water to retain moisture, then placed in a plant growth chamber and kept in the dark for 2 days. Another group of leaves was firstly incubated with mycelial plugs as above for testing the curative activity. The cell-free fermentation broth, carbendazim (800 mg/L), or sterile water were then sprayed onto the leaves after 24 h, and the leaves were cultured in the same conditions as above. The diameter of the *S. sclerotiorum* lesion was measured and calculating the inhibition rate was calculated as follows:

$$\mathrm{Inhibition}\mathrm{rate}\left(\%\right)=\frac{\text{A}\,c-\text{At}}{\text{A}\,t-0.5}\times 100$$

where Ac is the disease area diameter of the control, and At is the disease area diameter of the treatment. The diameter of the mycelial plug is 0.5 mm.

The antifungal inhibitory effects of metabolites of strain 135 on *G. graminis in vivo* were determined in pot experiments. The inoculum preparation of *G. graminis* followed the method of Durán et al. (2018) with a few modifications. Oat kernels were soaked in water for 24 h, then drained and mixed with the same amount of sand that had been washed with water. The mixture was sterilized at 121°C for 15 min on three consecutive days. Six agar plugs from a 7-day culture plate of *G. graminis* were applied into a 200 g sterile oat/sand mixture, then cultured for 20 days at 28°C to prepare the Ggt (*G. graminis*) inoculum.

Each pot contained 200 g soil and 16 wheat seeds (*Triticum aestivum* L.) (Mingxian-169); nine pots formed one treatment group. The protective and curative assays were conducted 7 days after seeding. The protective effect was determined by watering pots with either 50 mL fermentation broth as the test treatment, sterile water as the blank control, or difenoconazole (370 mg/L) as the positive control. After 24 h, the Ggt inoculum was applied at 10% related to soil weight per pot. The curative effect was determined by applying the Ggt inoculum at 10% related to soil weight per pot and incubating for 24 h, then watering with 50 mL fermentation broth, difenoconazole (370 mg/L), or sterile water. The disease index of take-all disease was determined according to Wilson et al. (1988) after 30 days. The control efficacy (%) was calculated as follows:

$$\mathrm{Inhibition}\mathrm{rate}\left(\%\right)=\frac{\text{B}\,c-\text{Bt}}{\text{Bt}}\times 100$$

where Bc is the disease index of the fungicide treatment, and Bt is the disease index of the blank treatment.

A pot experiment was also performed to evaluate the antifungal inhibitory effects of metabolites of strain 135 on *P. capsici* using 3-week-old pepper (*Capsicum annuum* L.) cultivar “Shijihong,” as described by Sang and Kim (2012) and Wang et al. (2016). Briefly, pepper seedlings were grown in 10 cm diameter pots containing 200 g soil for 3 weeks. The protective effect was determined by watering pots with either 50 mL fermentation broth as the test treatment, sterile water as the blank control, or metalaxyl (250 mg/L) as the positive control; there were nine pots per treatment. After 24 h, 50 mL of each bacterial suspension (10^8^ cells/mL) (*P. capsici*) was applied to each pot. The curative effect was determined by incubating seedlings with the bacterial suspension first and then watering with 50 mL fermentation broth, metalaxyl (250 mg/L), or sterile water 24 h later. The disease index was evaluated on a scale of 0 (symptomless) to 5 (plants dead) according to Kim et al. (Sang and Kim, 2012) after 14 days.

### Genomic DNA and Sequence Analysis

Genomic DNA of strain 135 was extracted using a QIAGEN Genomic-tip (QIAGEN Biotech Co., Ltd.) following the manufacturer’s instructions. Quality inspection of DNA purity, concentration, and integrity was performed by Thermo Scientific NanoDrop, Qubit, and 0.35% agarose gel electrophoresis, respectively, then a genomic DNA library was constructed using a Ligation Sequencing Kit (Oxford Nanopore Technologies Ltd) for Nanopore sequencing. The library was sequenced using a Promethion sequencer 48. Canu v1.5 (Koren et al., 2017) was used to assemble the filtered subreads and Pilon v1.22 (Walker et al., 2014) was used to correct the assembly sequence with higher accuracy. Sequencing work was conducted by Biomarker Technologies Co., Ltd.^1^

Secondary metabolite-related BGCs were identified using Antismash 5.0 (Blin et al., 2019). The PKS/NRPS analysis website^2^ was used for analysis and alignment of the domains related to biosynthesis of X-14952B (*vens* represent polyketide chain synthesis genes). Clustal X2 was used to align amino acid sequences.^3^

### Phylogenetic Analysis and Genome Comparison

Identification of actinomycetes at the genus level was fulfilled using polyphasic approaches, including culture, morphological, physiological, and biochemical characteristics, as described by Basilio et al. (2003). Morphological and cultural characteristics of the isolate were determined according to the International *Streptomyces* Project guidelines (Shirling and Gottlieb, 1966). Taxonomic identification was performed using 16S rRNA gene sequence analysis. After obtaining the assembled genome, the complete 16S rRNA gene sequence of strain 135 was isolated from the whole genome sequence and was aligned in the genus *Streptomyces* with representative sequences of related type strains retrieved from the GenBank/EMBL/DDBJ databases. Sequence alignments were adjusted manually before reconstructing phylogenetic trees with the neighbor-joining, maximum-likelihood, and maximum parsimony algorithms using MEGA X software (Kumar et al., 2018). Kimura’s two-parameter model was applied to compute the evolutionary distance for the maximum-likelihood and neighbor-joining phylogenetic trees (Nishimaki and Sato, 2019). Based on the 1,000 bootstrap resampling method of Felsenstein (1985), the resultant trees were evaluated for stability. Sequence similarity and DNA-DNA hybridization (DDH) values were calculated separately by Chun Lab’s online average nucleotide identity (ANI) Calculator^4^ and the Genome–Genome Distance Calculator GGDCv 2. 1 (Meier-Kolthoff et al., 2013) using all *Streptomyces* genomes as the reference.

Core, accessory, and unique pan-genome orthologous groups (POGs) were analyzed using the Bacterial Pan-Genome Analysis Tool (BPGA; Chaudhari et al., 2016). POGs were clustered with the USEARCH algorithm, setting the identity value to 0.5 for all 24 strains. The GGDC website^5^ was used to establish the phylogenomic tree of strain 135 and other closely related *Streptomyces* species. The pan-and core-genome tree was reconstructed with aligned amino acid sequences using MEGA X (Kumar et al., 2016) with the neighbor-joining algorithm and 1,000 bootstrap replications. The OrthoANI value was calculated between four closely related type strains (*Streptomyces huasconensis* HST28^T^, *Streptomyces alboniger* ATCC 12461^T^, *Streptomyces kanamyceticus* ATCC 12853^T^, and *Streptomyces formicae* KY5^T^) to strain 135 that formed a clade in the pan-and core-genome tree. Chun Lab’s Orthologous Average Nucleotide Identity Tool (OAT) (Lee et al., 2016) provided by EzBioCloud database was used for the calculation of OrthoAN values. All genome sequences were downloaded from the GenBank database.

### Fermentation and Isolation of Antimicrobial Metabolite From Strain 135

Large-scale fermentation of strain 135 was performed using 1L Erlenmeyer flasks containing 500 mL millet medium. Six agar plugs from an active-grown colony were transferred into each flask for 7 days at 28°C and 170 rpm. The whole culture (100 L) was centrifuged at 4°C, 8,000 rpm for 30 min.

The supernatant was concentrated into 10 L using rotary steaming at 40°C and extraction with an equal volume of chloroform; the process was repeated five times, each lasting 60 min. The organic phase was then centrifuged and evaporated to dryness using reduced pressure at 40°C. The solid, brownish-red residue (30 g) was resuspended in chloroform, mixed with an equal amount of silica gel (80-100 mesh) and applied to a silica gel column (200 mm × 900 mm i.d.) filled with 150 g silica gel (200–300 mesh) for chromatography. Chloroform-methanol (100:1, 60:1, 50:1, 20:1, 5:1, and 1:1) was used as the mobile phase to elute seven separate fractions named T1–T6. Fraction T2 was subsequently further separated by silica gel column chromatography and elution with petroleum ether ethyl acetate (100:1, 60:1, 20:1, 10:1, and 1:1); compound 1 (X-14952B, 32 mg) was obtained when the elution ratio was 10:1.

Structural identification of X-14952B was performed based on spectroscopic analysis. Nuclear magnetic resonance (NMR) spectra were recorded in methanol-D4 on a Bruker AVANCE III 500 spectrometer at room temperature. High-resolution electrospray ionization mass spectrometry (HRESIMS) spectra were recorded on a Mariner Mass 5, 304 instrument (CA, United States) or an AB SciexTripleTOF 5, 600 + System.

### Accession Number

Strain 135 is preserved at China General Microbiological Culture Collection Center (CGMCC) under isolate number No. 15109. The complete genome sequence of strain 135 and gene cluster sequences of X-14952B derived from strain 135 were submitted to NCBI with BioProject accession numbers PRJNA724811 and PRJNA724821, BioSmaple accession numbers SAMN18859273 and SAMN18863065, SRA accession numbers SRR14318471 and SRR 14318816, respectively. The genome GenBank accession number of strain 135 is CP075691.

## Results and Discussion

### Actinomycetes With Antimicrobial Activity

In total, 279 actinomycetes were isolated from 15 habitats. Qinghai Lake yielded the largest group of 83 actinomycete isolates, followed by Chaka with 79 isolates, while the Tuotuo river only yielded three isolates. Supplementary Table 1 shows the numbers and location information of actinomycetes isolated from different sampling sites.

Antifungal activities against *S. sclerotiorum* and *B. cinerea* of all 279 strains were tested by fermentation broth. The inhibition rates of 12 actinomycetes were greater than 70% on *S. sclerotiorum*, and 12 isolates had an inhibition rate greater than 60% on *B. cinerea* (Table 2), with some isolates featuring on both lists. Fermentation broth of strain 11-3-205 showed the highest activity against *S. sclerotiorum*, followed by strain 11-3-181 and strain 11-3-110. Strains 11-3-151, 11-3-177, and 11-3-96 all demonstrated good inhibition activity against *B. cinerea*. Overall, the inhibition rates of fermentation broth of the different isolates appeared disparate on all tested pathogenic fungi, meaning that fermentation broth with a strong inhibitory effect on one type of pathogenic fungi may not have the same strength on another type of pathogenic fungi. However, strain 135 could inhibit both types of pathogenic fungi and might therefore have a broad-spectrum antifungal activity to some extent. Consequently, strain 135 was selected for further investigation.

**TABLE 2:** Effect of different actinomycete isolates against two types of pathogenic fungi.

Strain	Inhibition rate for *Sclerotinia sclerotiorum* (%)	Strain	Inhibition rate for *Botrytis cinerea* (%)
11-3-205	99.05 ± 0.01	11-3-151	99.14 ± 0.01
11-3-181	98.26 ± 0.02	11-3-177	97.42 ± 0.00
11-3-110	95.57 ± 0.02	11-3-96	85.40 ± 0.08
11-3-106	91.32 ± 0.03	11-3-142	84.54 ± 0.07
11-3-85	90.02 ± 0.05	11-3-132	79.60 ± 0.01
11-3-194	86.55 ± 0.07	11-3-200	75.82 ± 0.16
11-3-237	84.72 ± 0.04	11-3-181	71.07 ± 0.03
11-2-130	83.59 ± 0.03	11-3-161	70.66 ± 0.11
11-3-207	80.56 ± 0.05	11-3-184	68.00 ± 0.13
11-3-114	74.83 ± 0.03	135	66.92 ± 0.15
135	73.35 ± 0.02	11-3-229	64.22 ± 0.01
11-3-203	72.92 ± 0.10	11-3-85	64.22 ± 0.10
Procymidone (500 mg/L)	98.26 ± 0.03	Procymidone (500 mg/L)	99.57 ± 0.02

*Data are presented as the average ± the standard deviation for three replicates.*

### Bioactivities of Strain 135 *in vitro*

To further explore the potential antimicrobial ability of strain 135, its inhibitory effects on 15 common plant-pathogenic fungi, two oomycetes, and seven bacteria were evaluated *in vitro*, and the results are shown in Tables 3, 4. Fermentation broth of strain 135 had strong inhibition effects on all tested pathogenic fungi and oomycetes, especially *S. sclerotiorum*, *B. cinerea*, *V. mali*, *P. capsici*, *G. cingulata*, *M. grisea*, *B. maydis*, and *E. turcicum*, where inhibition rates were greater than 70%. Metabolites of strain 135 also demonstrated strong antibacterial activity on *B. subtilis*, *P. syringae*, *Xanthomonas campestris*, *E carotovora*, and *S. aureus*, with similar inhibition activity as the streptomycin (1 mg/mL) positive control. These findings indicated that strain 135 had the ability to produce broad-spectrum antimicrobial metabolites.

**TABLE 3:** Inhibition effects of strain 135 against different plant pathogenic fungi and oomycetes.

Experimental pathogen	Inhibition rate (%)	Experimental pathogen	Inhibition rate (%)
*Rhizoctonia solani*	58.43 ± 0.87	*Fusarium graminearum*	51.10 ± 2.69
*Sclerotinia sclerotiorum*	72.10 ± 1.17	*Bipolaris maydis*	74.12 ± 1.61
*Botrytis cinerea*	73.26 ± 1.17	*Exserohilum turcicum*	79.29 ± 0.21
*Valsa mali*	100.00 ± 0.00	*Phytophthora infestans*	14.23 ± 0.51
*Phytophthora capsici*	75.96 ± 0.96	*Fusarium oxysporum*	23.95 ± 2.41
*Glomerella cingulata*	77.91 ± 0.00	*Gaeumannomyces graminis*	73.68 ± 0.54
*Magnaporthe grisea*	85.38 ± 0.44	*Verticillium dahliae*	25.60 ± 2.65
*Botrytis cinerea*	61. 82 ± 1. 27	*Alternaria solani*	61.96 ± 0.94

*Data are presented as the average ± the standard deviation for three replicates.*

**TABLE 4:** Antibacterial effect of strain 135.

Experimental pathogen	Inhibition diameter (mm)	
	**Fermentation broth**	**Streptomycin (1 mg/mL)**
*Bacillus subtilis*	27.34 ± 0.17	34.34 ± 0.17
*Pseudomonas syringae*	30.50 ± 0.76	38.67 ± 0.83
*Xanthomonas campestris*	27.00 ± 0.00	25.33 ± 0.33
*Erwinia carotovora*	19.00 ± 0.50	19.83 ± 0.44
*Xanthomonas juglandis*	21.00 ± 0.00	23.67 ± 0.17
*Staphylococcus aureus*	16.00 ± 0.29	21.17 ± 0.33
*Ralstonia solanacearum*	11.00 ± 0.00	13.17 ± 0.33

*The diameter of the bacteriostatic ring was subtracted from the diameter of the punch. Data are presented as the average ± the standard deviation for three replicates.*

### Effects of Fermentation Broth on Three Phytopathogenic Fungi *in vivo*

Experiments on detached rape leaves indicated that the fermentation broth of strain 135 displayed both protective and curative efficacy against *S. sclerotiorum*. The curative efficacy of fermentation broth of strain 135 (74.18%) was markedly higher than that of the positive control, carbendazim (800 mg/L) (36.27%) (Table 5), while the protective efficacy (54.04%) was similar to that of carbendazim (67.17%) (Supplementary Figure 1).

**TABLE 5:** Inhibitory effects of metabolites of strain 135 on *Sclerotinia sclerotiorum* (detached leaf method).

Treatment	Protective efficacy		Curative efficacy	
		
	Lesion diameter (cm)	Control efficacy (%)	Lesion diameter (cm)	Control efficacy (%)
Strain 135 fermentation broth	2.43 ± 1.83	54.04	1.68 ± 0.67	74.18
Carbendazim (800 mg/L)	1.88 ± 0.32	67.14	3.31 ± 1.49	36.27
Sterile water	4.70 ± 2.76		4.87 ± 2.32	

*For both protective and curative effects, the treatments are fermentation broth of *Streptomyces* sp. 135, positive control (carbendazim at 800 mg/L), and sterile water (blank control). Data are presented as the average ± the standard deviation for three replicates.*

In pot experiments with *G. graminis* in wheat, the protective efficacy of the fermentation broth was better than the curative efficacy. The protective effect of the fermentation broth of strain 135 was 90.66%, while that of the positive control, difenoconazole (370 mg/L), was 70.64%. However, difenoconazole (370 mg/L) had greater curative efficacy (93.48%) compared with the fermentation broth (67.99%) (Table 6 and Supplementary Figure 2). In contrast, in the pot experiment on *P. capsici*, the protective efficacy of the fermentation broth of strain 135 was slightly lower than that of the positive control (metalaxyl, 250 mg/mL) and the curative efficacy was similar to the positive control. The protective efficacies for the fermentation broth of strain 135 and the metalaxyl positive control were 79.33 and 84.33%, respectively, while the curative efficacies were 66.67 and 71.42%, respectively (Table 6 and Supplementary Figure 3).

**TABLE 6:** Inhibitory effects of metabolites of strain 135 on *Gaeumannomyces graminis* and *Phytophthora capsici* (pot experiments).

Pathogen	Treatment	Protective efficacy		Curative efficacy	
			
		Disease index	Control efficacy (%)	Disease index	Control efficacy (%)
***Gaeumannomyces graminis***					
	Strain 135 fermentation broth	7.35 ± 1.23	90.66	24.89 ± 2.75	67.99
	Difenoconazole (370 mg/L)	22.50 ± 3.03	70.64	4.87 ± 1.24	93.48
	Sterile water	76.68 ± 4.88		76.97 ± 3.38	
***Phytophthora capsici***					
	Strain 135 fermentation broth	16.00 ± 7.48	79.33	28.00 ± 4.90	66.67
	Metalaxyl (250 mg/mL)	12.00 ± 4.90	84.33	24.00 ± 4.00	71.42
	Sterile water	80.00 ± 6.32		84.00 ± 7.48	

*For both protective and curative effects, the treatments are fermentation broth of *Streptomyces* sp. 135, positive control (difenoconazole at 370 mg/L for *Gaeumannomyces graminis* and metalaxyl at 250 mg/mL for *Phytophthora capsici*), and sterile water (blank control). Data are presented as the average ± the standard deviation for three replicates.*

These findings indicated that **s**train 135 had a good biocontrol efficacy against fungal disease caused by *S. sclerotiorum*, *P. capsici*, and *G. graminis*. Therefore, strain 135 might be an excellent antimicrobial agent for controlling agricultural fungal and bacterial diseases.

### Antimicrobial Activity of X-14952B

The antimicrobial activities of X-14952B against seven plant-pathogenic fungi and six bacteria were evaluated, including *S. sclerotiorum*, *A. alternata*, *G. cingulata*, *F. graminearum*, *P. capsici*, *G. graminis*, *B. cinerea*, *B. subtilis*, *E. carotovora*, *R. solanacearum*, *P. syringae*, *Xanthomonas oryzae*, and *X. citri*, which infect the main food and economic crops (Tables 7, 8, and Supplementary Figures 4, 5). There are limited reports on X-14952B since 1985, and results from the current study were the same as previous reports (Omura et al., 1985; Fourati-Ben Fguira et al., 2005; Tian et al., 2017), with X-14952B exhibiting higher inhibition rates against fungi than against bacteria. Among the tested pathogenic fungi, X-14952B showed the greatest antifungal activity against *A. alternata*, *G. graminis*, *B. cinerea*, and especially *S. sclerotiorum* and *F. graminearum*, for which it had a 50% inhibition concentration (EC50) of up to 0.049 and 0.04 μg/mL, respectively (Table 7). For some pathogens, the antibacterial activity of X-14952B was higher than that of the positive control such as *R. solanacearum*, the MIC of X-14952B was up to 12.25 μg/mL and the MIC of the positive control, Streptomycin, is 31.25 μg/mL (Table 8). In summary, these results indicated that X-14952B was a broad-spectrum antimicrobial metabolite and has potential as an antifungal agent.

**TABLE 7:** Antifungal effect of X-14952B.

Compound	50% inhibition concentration (EC50) (μg/mL)							
	Ss	Aa	Gc	Fg	Pc	Gg	Bc	Tc
X-14952B	0.049	0.2	6.21	0.04	5.74	0.61	0.574	3.0
Carbendazol	0.06	>100	0.067	0.5	2.87	0.52	1.138	0.2

*Ss, *Sclerotinia sclerotiorum*; Aa, *Alternaria alternata*; Gc, *Glomerella cingulata*; Fg, *Fusarium graminearum*; Pc, *Phytophthora capsic*; Gg, *Gaeumannomyces graminis*; Bc, *Botrytis cinerea*; Tc, *Thanatephorus cucumeris.**

**TABLE 8:** Antibacterial effect of X-14952B.

Pathogenic bacteria	Minimum inhibitory concentration (MIC) (μg/mL)	
	
	X-14952B	Streptomycin
*Pseudomonas syringae*	50	1.56
*Xanthomonas oryzae*	25	3.25
*Ralstonia solanacearum*	12.5	31.25
*Bacillus subtilis*	12.5	2.5
*Erwinia carotovora*	>400	>400
*Xanthomonas citri*	>400	>400

*Cultured at 28°C for 24 h.*

### Genome Sequencing, Comparative and Phylogenetic Analysis of Strain 135

The complete 16S rRNA gene sequence of strain 135 was obtained from the genome and uploaded to the Ezbiocloud database. Sequence similarities between strain 135 and all other model species with a total of 42 different species of the genus *Streptomyces* ranged from 96.98 to 98.95%. The 16S rRNA gene sequence similarities of strain 135 with members of other genera were less than 90%, indicating that strain 135 belonged to the genus *Streptomyces*.

**FIGURE 1:**
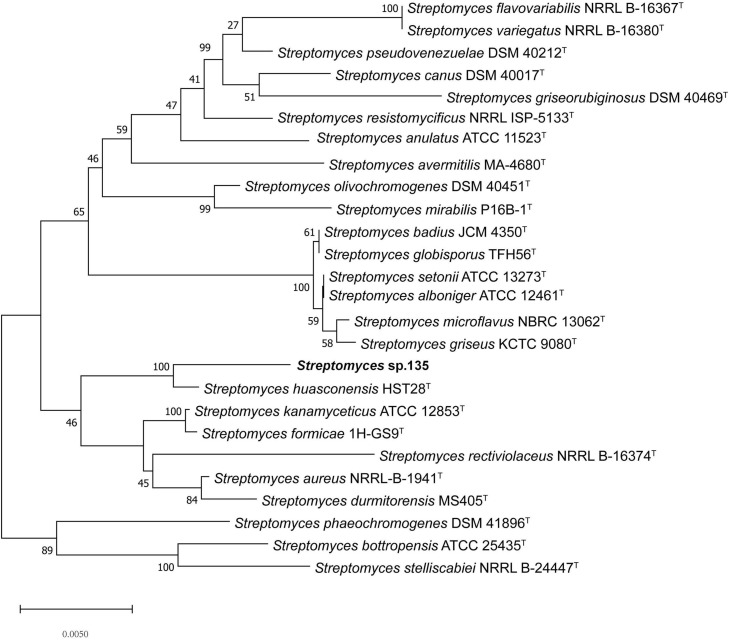
Phylogenetic analysis of strain 135 using complete 16S rRNA gene sequences (1,441 nucleotides) and the neighbor-joining algorithm. Numbers at nodes represent the percentage of 1,000 bootstrap resamples; Bar, 0.005 substitutions per site. The complete 16S rRNA gene sequences of all type trains and strain 135 was isolated from the whole genome sequence. Sequence alignments were adjusted. GenBank assembly genome accession numbers of all strains used in this study were list as below except strain 135: *Streptomyces alboniger* ATCC 12461^T^: CP023695.1 *Streptomyces aureus* NRRL-B-1941^T^: BA000030.4 *Streptomyces avermitilis* MA-4680^T^: BA000030.4 *Streptomyces badius* JCM4350^T^: NZ_LWMP01000001.1 *Streptomyces bottropensis* ATCC 25435^T^: KB911581.1 *Streptomyces canus* DSM 40017^T^: KQ948656.1 *Streptomyces flavovariabilis* NRRL B-16367^T^: JNXD01000001.1 *Streptomyces formicae* 1H-GS9^T^: NZ_CP022685.1 *Streptomyces globisporus* TFH56^T^: CP029361.1 *Streptomyces durmitorensis* MS405^T^: NR_043520.1 *Streptomyces griseorubiginosus* DSM 40469^T^: CP032427.1 *Streptomyces griseus* ATCC 13273^T^: NZ_CP032543.1 *Streptomyces huasconensis* HST28^T^: NZ_RBWT00000000. 1 *Streptomyces kanamyceticus* ATCC 12852^T^: CP029361.1 *Streptomyces microflavus* NBRC 13062^T^: BMUG01000001.1 *Streptomyces mirabilis* P16B-1^T^: NZ_KN050734.1 *Streptomyces olivochromogenes* DSM 40451^T^: KQ948451.1 *Streptomyces phaeoluteigriseus* DSM 41896^T^: MPOH02000001.1 *Streptomyces pseudovenezuelae* DSM 40212^T^: KQ948144.1 *Streptomyces rectiviolaceus* NRRL B-16374^T^: NR_043502.1 *Streptomyces resistomycificus* NRRL ISP-5133^T^: KQ948988.1 *Streptomyces setonii* NRRL ATCC 13273^T^: CP032543.1 *Streptomyces stelliscabiei* NRRL B-24447^T^: LBNW01000001.1 *Streptomyces variegatus* NRRL B-16380^T^: JYJH01000001.1 *Streptomyces kanamyceticus* ATCC 12852^T^: NZ_CP023699.1.

To ascertain the phylogenetic position of strain 135, phylogenetic trees were reconstructed and revealed *S. huasconensis* HST28^T^ as the closest relative to strain 135 with a 16S rRNA gene sequence similarity of 98.96% and clustering in the same clade with high bootstrap values in the neighbor-joining (1,000 bootstrap value, Figure 1), maximum-likelihood (94, Supplementary Figure 6), and maximum-parsimony (97, Supplementary Figure 7) trees.

Based on GenBank/EMBL/DDBJ databases database, we preliminarily chose 23 strains together with strain 135 to analyzed the pan-genome (*Streptomyces bottropensis* ATCC 25435^T^, *Streptomyces stelliscabiei* NRRL B-24447^T^, *Streptomyces aureus* NRRL-B-1941^T^, *S. formicae* 1H-GS9^T^, *S. huasconensis* HST28^T^, *Streptomyces badius* JCM4350^T^, *Streptomyces globisporus* TFH56^T^, *Streptomyces microflavus* NBRC 13062^T^, *Streptomyces anulatus* ATCC 11523^T^, *Streptomyces setonii* ATCC 13273^T^, *Streptomyces flavovariabilis* NRRL B-16367^T^, *Streptomyces variegatus* NRRL B-16380^T^, *Streptomyces pseudovenezuelae* DSM 40212^T^, *S. alboniger* NRRL B-16380^T^, *Streptomyces griseus* ATCC 13273^T^, *Streptomyces phaeoluteigriseus* DSM 41896^T^, *Streptomyces resistomycificus* NRRL ISP-5133^T^, *Streptomyces canus* DSM 40017^T^, *Streptomyces griseorubiginosus* DSM 40469^T^, *Streptomyces avermitilis* MA-4680^T^, *Streptomyces mirabilis* NBRC 1345^T^, *Streptomyces olivochromogenes* DSM 40451^T^, and *S. kanamyceticus* ATCC 12852^T^). The results showed that strain 135 shared homologous coding sequences (CDSs) and clustered into the core genome of *Streptomyces*, which is consistent with the previous results. Therefore, we constructed a core-genome tree based on these core homologous proteins of all 24 strains (Supplementary Figure 8). The total number of genes in the whole genome of *Streptomyces* showed an upward trend indicating that the whole genome is open. The progression of the pan- and core-genomes are shown in Supplementary Figure 9.

The most core, accessory and unique POGs were related to general function, transcription and metabolism and so on (Supplementary Figure 10). Pan-genomic analysis shows that strain 135 and four related species formed in the same clade of core genome phylogenetic tree which had 7,287 POGs: 1,704 POG core, 4,719 POG accessory, and 851 unique POGs, their number of unique and core POGs are displayed in Supplementary Figure 11.

Unlike the phylogenetic tree of 16S rRNA, the core genome tree shows *S. huasconensis* HST28^T^ has a longer branch compared with strain 135, which means it has a more complex genomes composition (Figure 1 and Supplementary Figure 8). The two phylogenetic tree shows slight difference compared with each other. In 16S rRNA phylogenetic tree, *S. aureus* NRRL B-1941^T^ formed in the same cluster of strain 135. But in the core genome tree, *S. aureus* NRRL B-1941^T^ is on another cluster of the evolutionary tree, and *S. alboniger* ATCC 12461^T^ together with *S. formicae* 1H-GS9^T^ are incorporated in the clade of strain 135. The pan-genome tree and the phylogenomic tree (Supplementary Figures 12, 13) show the same situation with others. Whether in 16S rRNA phylogenetic tree or core-,pan-genome tree and the phylogenomic tree, *Streptomyces* sp. 135 are formed the same clade of *S. huasconensis* HST28^T^ which illustrates that they are closely related species. Meanwhile, we selected type strains in the core genome tree which in the same cluster of strain 135 and strains in other clusters to calculate the gene distance of DDH and ANI values. DDH and ANI values of strain 135 with the selected type strains varied from 20.9 to 56.5 and 75.79 to 91.39%, respectively (Table 9), which are substantially less than the demarcation points of 70% (DDH) and 95% (ANI). Then we selected type strains in the core-and pan-genome tree which in the same cluster of strain 135. Similarly, OrthoANI values (Table 9) are less than the demarcation points of 95%. These three index reported as thresholds to delineate bacterial species (Meier-Kolthoff et al., 2013; Lee et al., 2016; Olm et al., 2020). These data revealed the possibility that strain 135 might be a potential novel species of the genus *Streptomyces* but need further verifications.

**TABLE 9:** Average nucleotide identity, dDDH, and OrthoANI values of strain 135 with other members of the genus *Streptomyces*.

Strains				Strain 135	
		
				ANI (%)	dDDH (%)
*Streptomyces griseus* KCTC 9080^T^ NC_010572. 1				78.62	21.8
*Streptomyces canus* DSM 40017^T^ NZ_LMWU00000000. 1				79.23	l21.1
*Streptomyces formicae* 1H-GS9^T^ NZ_CP022685. 1				85.71	38.3
*Streptomyces alboniger* NRRLB-16380^T^ NZ_CP023695. 1				88.76	49.3
*Streptomyces huasconensis* HST28^T^ NZ_RBWT00000000. 1;				91.39	56.5
*Streptomyces aureus* NBRC 100912^T^ NZ_JNZD00000000. 1				79.37	21.3
*Streptomyces badius* NRRLB-2567^T^ NZ_LWMQ00000000. 1;				78.91	21.9
*Streptomyces anulatus* NRRLB-2000^T^ NZ_JPZP00000000. 1				78.88	21.6
*Streptomyces variegatus* NRRL B-16380^T^ NZ_JYJH00000000.1				75.79	23.8
*Streptomyces kanamyceticus* ATCC 12853^T^ NZ_CP023699.1				85.97	36.5
*Streptomyces flavovariabilis* NRRL B-16367^T^ NZ_JNXD00000000.1				79.56	23.8
*Streptomyces phaeoluteigriseus* DSM 41896^T^ NZ_MPOH00000000.2				79.58	22.5
*Streptomyces stelliscabiei* NRRL B-24447^T^ NZ_JPPZ00000000.1				79.72	20.9
*Streptomyces bottropensis* ATCC 25435^T^ NZ_KB911581.1				79.64	21.6

**Strain**	**Sk**	**Sh (%)**	**Sa (%)**	**Sf (%)**	***Streptomyces*** **sp. 135 (%)**

**OrthoANI values**					
Sk		83.95	84.46	93.49	85.97
Sh			88.94	89.53	91.74
Sa				86.46	89.13
Sf					85.99
*Streptomyces* sp. 135					

*OrthoANI values calculated using the OAT software between strain 135 and other closely related species in the same cluster of pan- and core-genome trees Sk, *Streptomyces kanamyceticus* ATCC 12853^*T*^; Sh, *Streptomyces huasconensis* HST28^*T*^; Sa, *Streptomyces alboniger* NRRLB-16380^*T*^; Sf, *Streptomyces formicae* 1H-GS9^*T*^.*

Ribosomal genes are essential for all living species and 16S rRNA genes are highly conserved to the same genus of bacteria, thus it was suggested as a useful tool in species identification. However, a single gene cannot reflect the subtle divergence of genomes and differentiate unrelated isolates, pan-genome tree and core genes play a vital part in filling the gap in these aspects (Leekitcharoenphon et al., 2012).

Phenotypic characteristics between strain 135 and *S. huasconensis* HST28^T^ were compared. There were significant differences in cultural characteristics of strain 135 and *S. huasconensis* HST28^T^, including the color of substrate mycelium and aerial mycelium when separately cultured in ISP3, ISP4, ISP5, and ISP7 media (Table 10). Strain 135 also exhibited marked differences in carbon source utilization compared with *S. huasconensis* HST28^T^ (Table 10). Strain 135 could utilize several carbon sources and organic acids unlike *S. huasconensis* HST28^T^. Strain 135 showed lower pH and salt tolerance compared to *S. huasconensis* HST28^T^, which also showed tolerance to some inhibitory compounds like vancomycin, tetrazolium violet, and tetrazolium blue (Table 10). These results demonstrate the uniqueness of strain 135.

**TABLE 10:** Growth and cultural features of *Streptomyces* strain 135.

Culture condition				

Media	*Streptomyces* sp. 135		*Streptomyces huasconensis HST28^T^*	
		
	Substrate mycelium color	Aerial mycelium color	Substrate mycelium color	Aerial mycelium color
ISP3	White	White	Grayish-green	Pale green
ISP4	Dark yellow	White	Strong yellowish-brown	White
ISP5	Dark yellow	Yellowish-brown	Moderate yellow	Yellowish-white
ISP7	Dark yellow	White	Dark yellow	Greenish-white

**Carbon source utilization**				
D-Turanose, α-D-Lactose, β-Methyl-D-Glucoside, D-Salicin, N-Acetyl-β-D-Mannosamine,			–	+
D-Fructose			+	w
D-Trehalose, D-Melibiose, N-Acetyl-D-Glucosamine, α-D-Glucose, D-Mannose, D-Galactose, L-Rhamnose, D-Mannitol, Glycerol			–	+
**Amino acids**				
D-Aspartic acid, Glycyl-L-proline,			w	+
D-Fructose-6-PO4			+	w
L-Arginine			+	+
L-Histidine, L-Pyroglutamic acid			w	+
**Organic acids**				
D-Glucuronic acid, β-Hydroxy-D,L-butyric acid			–	+
Methyl pyruvate			+	w
Acetoacetic acid			+	w
L-Galactonic acid lactone, p_Hydroxyphenylacetic acid, L-Lactic acid			–	+
**Inhibitory compounds**				
1 % Sodium lactate			–	+
Potassium tellurite			+	w
pH 5			w	–
pH 6			w	+
8% NaCl			w	–
D-Serine			+	–
Lithium chloride, Aztreonam			w	–
Vancomycin, Tetrazolium violet, Tetrazolium blue			w	+
Sodium bromate			–	+

*Cultured at 28°C for 7 days. +, positive; –, negative; w, borderline.*

Although the phylogenetic and phenotypic results described above indicated that strain 135 might be a potential novel species of the genus *Streptomyces*, identification of a strain to the species level requires these data to be combined with chemotaxonomic and morphology characteristics. Thus, future research to determine the chemotaxonomic and morphological characteristics of strain 135 should be conducted to characterize strain 135 below the taxonomic level of the genus.

The complete sequenced genome of strain 135 reveals a linear chromosome of 8,937,250 bp, and G+C content is 71.44%, which is within the normal range of the genus *Streptomyces* (60.1–71.9%) (Wright and Bibb, 1992). Whole-genome sequencing of strain 135 produced 599,074 reads, 2 contigs and 2 scaffolds, 8,056 protein-coding genes, 71 tRNA-coding genes, 18 rRNA-coding genes, and 40 other ncRNA genes. The average CDS length was 956 bp, and the coding density was approximately 88.25%. Compared with *S. huasconensis* HST28^T^, the genome size and G+C content of the two strains were very similar. However, the numbers of RNA and CDS were not the same. Strain 135 had more RNA and minor CDS in comparison with *S. huasconensis* HST28^T^. Specifically, *S. huasconensis* HST28^T^ has a genome size of 8.6 Mb, 71.5% G+C content, 67 RNAs, and 8,211 CDS (Cortés-Albayay et al., 2019). Within strain 135, a total of 129 RNA and 7,791 gene numbers were predicted (Supplementary Table 2).

### Detection of Antibiotic and Secondary Metabolite-Related Gene Clusters

Online antiSMASH software analysis of secondary metabolite BGCs identified 37 BGCs, including 9 nonribosomal peptide synthetases (NRPSs), 6 T1PKS, 1 T2PKS, 3 T3PKS, 3 terpenes, 2 lanthipeptide, 2 lasso peptide, 1 siderophore, 2 betalactone, 1 CDPS (cyclodipeptide synthases), 1 butyrolactone, 2 bacteriocins, 1 thiopeptide, and 3 other types of BGCs (Supplementary Table 3). As the BGC of X-14952B has not yet been reported, each gene cluster was analyzed, leading to the conclusion that cluster 10 contained either modular PKS genes or genes identified as components of BGCs for complex macrolides, which means cluster 10 has the possible function to encode X-14952B.

### Structure Elucidation of X-14952B and Related Gene Cluster Analysis

Through analysis of spectra from NMR spectroscopy and HRESIMS and comparison with published spectroscopic data, compound 1 was identified as the known compound X-14952B (Supplementary Table 4 and Supplementary Figures 14–17).

**FIGURE 2:**
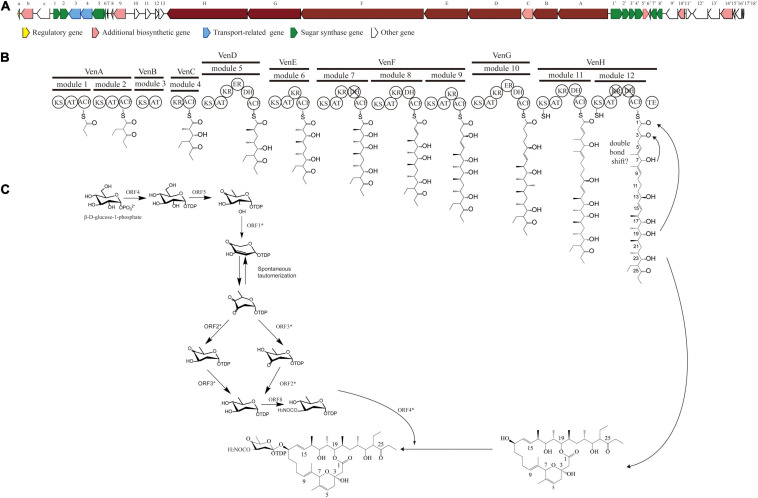
Gene clusters for X-14952B and its putative biosynthetic process. **(A)** Biosynthetic gene cluster (BGC) for X-14952B. **(B)** Deduced synthesis process of polyketide backbone and **(C)** sugar unit.

**FIGURE 3:**
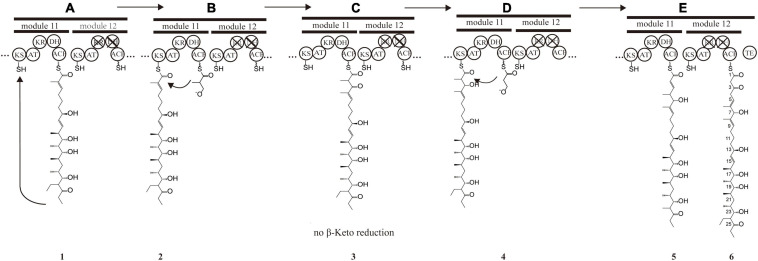
Presumptive model of stuttering process in module 11 and 12. **(A)** Polyketide chain 1 is “stuttering” after a normal extension. **(B)** Polyketide chain 1 is incorporated with one extender unit malonyl-CoA transformed into the polyketide chain 1. **(C)** β-keto at position C_7 in polyketide chain 2 does not reduce, and DH_11-mediated dehydration does not happen, forming polyketide chain 3. **(D)** Polyketide chain 3 is incorporated with one extender unit malonyl-CoA transformed into polyketide chain 5. **(E)** Polyketide chain 5 is incorporated with one extender unit malonyl-CoA without reduction and dehydration due to the inactivation of KR_12 and DH_12.

The PKS of X-14952B is encoded by eight genes (*venA* to *venH*) and contains 12 modules organized for biosynthetic assembly, including 50 enzymatic domains comprising one module for loading and 11 modules for expansion. The core domains, ketosynthase (KS), acyltransferase (AT), and acyl-carrier protein (ACP), are responsible for chain extension, and the optional parts – ketoreductase (KR), dehydratase (DH), and enoyl reductase (ER) – play a role in modifying the structure of the polyketide chain. Generally, the basic structure of a PKS can be predicted from the modular system of PKS since there is collinearity between the number of modules and the number of chain extensions. However, the backbone size of X-14952B is not consistent with the final products of the module domain. Iterative use of one or more modules will often result in longer polyketide chains than those predicted by the co-linear model (Chen and Du, 2016). The whole proposed biosynthesis process of X-14952B is shown in Figure 2. After analysis, some modules of X-14952B are predicted to be used iteratively in biosynthesis (Figure 3).

For X-14952B, module 1 to module 9 might be responsible for assembling the backbone from C_10 to C_27. Suppose the following extension steps guided by module 11 and module 12 expand only one time. In that case, the skeleton of the produced compound merely has 19 carbons, which means module 11 or module 12 undergoes iterated use at least twice. KR_12 and DH_12 were also found to be inactive. Hence the double bond between C_4 and C_5 must be formed by module 11. That is, module 11 has been used iteratively at least twice. The possible biosynthesis process of the carbon skeleton from C_1 to C_10 may follow these steps (Figure 3): after the first elongation in module 11, the acyl chain 1 is transferred from ACP_11 to KS_11, termed stuttering (A). Stuttering is uncommon in polyketide biosynthesis but does occur, such as in the biosynthesis process of erythromycin (Peng et al., 2019). Previous reports have explained the formation of this kind of compound, which is two carbons longer than the PKS structure (Tang et al., 2004). Next, one extender unit malonyl-CoA is incorporated into product 1 (B). The B1 type KR_11, although it has a “VDD” motif instead of “LDD” and this replacement occasionally appears in other active KRs like NYS 3 and NYS 12 from Nystatin (Brautaset et al., 2000), does not perform its function and this could result from the insufficient quantity of NADPH or a slow turnover rate. Therefore β-keto at C_7 position does not reduce, and naturally, DH_11-mediated dehydration cannot happen, leading to product 3 formation (C). KR_8 also has a “VDD” motif. However, according to the presumed synthesis process and the compound structure, the “VDD” motif does not influence the reductive activity because of the existence of the catalytic Lys, Tyr, and Ser moieties. Similar situations are present in the biosynthesis process of luteoreticulin (Peng et al., 2019). Product 3 is transferred backward from ACP_11 to KS_11, with no elongation, but β-ketoreduction and the dehydration step at the C_6 position take place as expected. KS_12 acts as a gatekeeper and does not accept the transfer of product 4 because it is not the correct size. Thus KS_11 receives 4 from its cognate ACP_11. Moreover, AT_11 selectively recognizes and incorporates malonyl rather than methylmalonyl (D). After two rounds of elongations, product 6 is released from the PKS.

In the separation process, strain 135 yields little X-14952B compared to other reports (Fourati-Ben Fguira et al., 2005; Tian et al., 2017). Compounds with a very similar polarity to X-14952B were not obtained, which indicated that X-14952B might be synthesized using modules iteratively. The complex polyketides core structure is built by repeated condensation of acyl-CoA. The AT domain is the “gatekeeper” of the module, which recognizes the specific elongation unit to be incorporated into the growing polyketide chain, and covalently connects the malonyl and methylmalonyl derivative of the elongation unit to the sulfhydryl group of the ACP prosthetic group. All AT domains were compared to analyze conserved motifs and active amino acid residues with substrate specificity. The AT domains of modules 1-12, except AT_2, AT_8, and AT_9, contain the “RDDVVQ” motif (residues 60-65) and “YASH” motif (residues 203-205), which are correlated with methylmalonyl-CoA specificity (Figure 3). Module 2 has the “FASH” motif, responsible for incorporating units from ethylmalonyl (EM)-CoA and other bulky CoA esters (Del Vecchio et al., 2003). This prediction is matched with the structure of X-14952B. EM units can be synthesized from primary or secondary metabolism (Jiang et al., 2015). Modules 8 and 9 contain the “QASH” motif (residues 203-205) and the “TVYTQT” motif similar to venturicidin A. These two modules might incorporate the units of malonyl-CoA. The planer structure matched perfectly with the predicted result except for the domains AT_10, AT_11, and AT_12 incorporating malonyl rather than methylmalonyl (Figure 2). Though AT domains have a strict specificity for a single acyl CoA substrate, some AT domains can bind at least two different monomers with similar efficiency (Reeves et al., 2001).

Ketoreductase domains play an essential role in setting stereocenters in polyketide biosynthesis. KR domains have been classified by the β-hydroxy stereochemistry of their products: the characteristic lack of “LDD” motif and the presence of highly conserved tryptophan, namely A-type KRs, which can yield an “S” hydroxyl. For B-type KRs, the “LDD” motif is indispensable. If the a-substituents are not epimerized or if a-substituent is epimerized while KRs with reducing activity, these kinds of KRs are called C-type (Keatinge-Clay, 2007). KRs cannot be described entirely as type A, type B, or type C, rather they are subdivided into six subtypes. KRs of X-14952B are sorted into three classes, namely A1 type, B1 type, and B2 type (Supplementary Figure 18), resulting in “R, S,” “R, R,” and “S, R” configurations. The KR_9 domain, classified as A1 type, determined the absolute configuration of the hydroxyl in C_13, given α, β-stereocenters with “R, S” configuration in the hydroxyl of C_13. The KR domain of B1 type, including KR_5, KR_7, and KR_11, form the “R, R”-configured α, β-stereocenters in C_20, C_16/17, C_8, and C_5/6. KR_4 and KR_6 belong to the B2 type KR domain, giving C_22/23 and C_18/19 α, β-stereocenters with “S, R”-configurations. The KR_12 domain is inoperative as it is missing the conserved “SXAGX” motif. The analysis of absolute configurations with the prediction by KR domain is consistent with the closely related congeners (Brufani et al., 1971).

In all six domains (DH_5, DH_8, DH_10, DH_11, DH_12), DH_7 and DH_12 are inactive although they contain the conserved “HXXGXXXP” motif. However, the “YPG” motif is absent, so could not facilitate in binding the b-hydroxyl group. Like venturicidin A, a double bond shift has occurred from C4/5 to C5/6. Olefin migration is uncommon in natural polyketide products such as Epothilone and Ansamitocin (Tang et al., 2004; Taft et al., 2009). The apparent ability to catalyze additional or alternate dehydration can only be inferred because of the lack of crystal structure or low crystal structure of the DH domains (Taft et al., 2009).

The two ER domains, that is, ER_5 and ER_10, are both active since the existence of the “LXHXg (a)XGGVG” sequence correlates with NADP (H) binding (Ruan et al., 1997).

Genes associated with the biosynthesis of sugar motifs are homologous with concanavalin A on deoxysugar biosynthesis (Haydock et al., 2005) and with the exact progress of venturicidin A (Li et al., 2021). The initial step of sugar biosynthesis is similar to those of other deoxysugars with the activation of glucose through glucose-1-phosphate thymidylyltransferase (ORF4) (Table 11) converting it into TDP-D-glucose, then the 4, 6-dehydration of TDP-glucose controlled by dTDP-glucose 4, 6-dehydratase (ORF5)to produce TDP-4-keto-6-deoxyglucose. To yield TDP-3, 4-diketo-2, 6-dideoxy-D-glucose, C-2 nation deoxygenation is controlled by NDP-hexose 2, 3-dehydratase (ORF1^∗^), producing the 2, 4-diketo derivative, which can spontaneously transform into TDP-3, 4-diketo-2, 6-dideoxy-D-glucose. Then 4- (ORF3^∗^) and 3-ketoreductases (ORF2^∗^) directly reduce each keto group twice respectively on C3 and C4 to obtain 2-deoxy derivatives with appropriate stereochemistry at C3 and C4 positions. Carbamoyltransferase (ORF8) catalyze 3-O-carbamoylation to generate 3′-O-carbamoyl-2′-deoxy-D-rhamnoside. Like *venE* encoding venturicidin A, ORF8 might encode TDP-d-olive 3-O-carbamoylation, which is different from known carbamoyltransferases represented for 4-O-carbamoylation, like *con7* of concanamycin biosynthesis and *novN* of novobiocin biosynthesis (Thibodeaux et al., 2008). In the final process, through glycosyltransferase (ORF4^∗^), the sugar moiety is transmitted to the polyketide scaffold. The critical genes related to the synthesis of X-14952B are listed in Table 11.

**TABLE 11:** Deduced functions of ORFs in *ven* biosynthetic gene clusters (BGCs).

Protein	Size (aa)	Protein homolog and origin	Identity (%)	Proposed function
4	292	*Streptomyces*	89.24	Glucose-1-phosphate thymidylyltransferase
5	324	*Streptomyces*	92	dTDP-glucose 4, 6-dehydratase
6	513	*Streptomyces luteocolor*	78	ABC transporter ATP-binding protein
7	574	*Streptomyces sparsogenes*	78	ABC transporter ATP-binding protein
8	597	*Streptomyces*	92.13	Carbamoyltransferase
12	637	*Streptomyces* sp. NRRL S-4	87.07	Type I polyketide synthase
H	3637	*Streptomyces* sp. NRRL S-4	87.3	Type I polyketide synthase
G	2183	*Streptomyces luteocolor*	88.69	Type I polyketide synthase
F	5172	*Streptomyces silaceus*	85.57	Type I polyketide synthase
E	1710	*Streptomyces* sp. NRRL S-4	89.25	Type I polyketide synthase
D	2047	*Streptomyces sparsogenes*	84.13	Type I polyketide synthase
C	366	*Streptomyces silaceus*	86.45	Polyketide synthase
B	1038	*Streptomyces silaceus*	83.84	Type I polyketide synthase
A	2157	*Streptomyces* sp. NRRL S-4	80.39	Type I polyketide synthase
1*	499	*Streptomyces*	84.17	NDP-hexose 2, 3-dehydratase
2*	344	*Streptomyces* sp. NRRL S-4	79.59	3-ketoreductase
3*	245	*Streptomyces* sp. NRRL F-5650	77.14	4-ketoreductase
4*	417	*Streptomyces*	92.57	6-deoxy-D-allosyltransferase
5*	248	*Streptomyces silaceus*	85.89	Thioesterase

There are some regulatory genes in cluster 10 that are involved in amino acids, sugar transport and metabolism, signal transduction, gene regulation, and some unknown functions (Table 12). For genome mining tools, it is challenging to establish the boundaries of BGCs. Antismash, the genomic analysis tool used in the current study, contains as many genes as possible to ensure functional integrity (Blin et al., 2017). Thus, some margin areas might be predicted in the gene clusters. However, in terms of the synthesis process, the edge of the BGC can only be accurately determined by systematic gene destruction and analysis of the resulting phenotype. Further research using such techniques is hoped to clarify the role of these genes in the future.

**TABLE 12:** Possible margin ORFs in *ven* biosynthetic gene clusters (BGCs).

Protein	Size (aa)	Protein homolog and origin	Identity (%)	Proposed function
a	151	*Streptomyces*	97	Putative_transcription_regulator
b	463	*Streptomyces* sp. BA2	97	Amino acid permease
c	522	*Streptomyces* sp. G44	98	Putative_inosine_monophosphate_dehydrogenase
1	151	*Streptomyces*	97	Transcription_regulator
2	463	*Streptomyces* sp. BA2	97	Amino acid permease
3	522	*Streptomyces* sp. G44	98	Puinosine_monophosphate_dehydrogenase
9	106	*Streptomyces luteocolor*	77	Beta fold hydrolase
10	30	*Streptomyces* sp. SID10244	69	AMP-binding protein
11	81	*Streptomyces* sp. NRRL S-15	84.31	Acyltransferase domain-containing protein
13	149	*Streptomyces* sp. ADI91-18	52	Hypothetical protein
14	177	*Streptomyces rimosus*	49	Hypothetical protein
15	103	*Streptomyces* sp. ADI95-17	58	Hypothetical protein
16	191	*Streptomyces* sp. ADI91-18	80.36	Hypothetical protein
6*	123	*Streptomyces* sp. G44	84.55	Barstar family protein
7*	332	*Streptomyces alboniger*	99.7	Sugar-binding domain-containing protein
8*	228	*Streptomyces* sp. G44	97.84	Ribulose-phosphate 3-epimese
9*	486	*Streptomyces* sp. WAC 01529	95.06	rRNA cytosine-C5-methyltransferase
10*	314	*Streptomyces* sp. WAC 01529	97.12	Methionyl-tRNAformyltransferase
11*	186	*Streptomyces* sp. G44	94.62	Hypothetical protein
12*	726	*Streptomyces* sp. NRRL S-920	94.63	Primosomal protein N
13*	439	*Streptomyces atriruber*	99.5	Methionine adenosyltransferase
14*	414	*Streptomyces venezuelae*	96.37	Bifunctional phosphopantothenoylcysteine decarboxylase/phosphopantothenate–cysteine ligase CoaBC
15*	90	*Streptomyces* sp. YIM 121038]	100	DNA-directed RNA polymerase subunit omega
16*	197	*Streptomyces*	99.49	Guanylate kinase
17*	53	*Streptomyces*	100	Integration host factor
18*	33	*Streptomyces* sp. SID8455	100	Integration host factor

## Conclusion

In this study, an X-14952B-producing strain, strain 135 isolated from Qinghai-Tibet Plateau with broad antimicrobial activity was tested *in vitro* using seventeen fungi, two oomycetes, and six bacteria, and *in vivo* using three fungi which illustrated strain 135 might be an excellent antimicrobial agent for controlling agricultural fungal diseases. Then the genomic analysis and morphological and phylogenetic characterization suggested that strain 135 belonged to the genus *Streptomyces*. Cultural characteristics together with DDH, ANI and the OrthoANI values revealed that strain 135 could be distinguished from its closely related strains. Furthermore, the gene cluster and biosynthetic pathway of X-14952B were analyzed and inferred by whole-genome analysis.

## Data Availability Statement

The datasets presented in this study can be found in online repositories. The names of the repository/repositories and accession number(s) can be found below: https://www.ncbi.nlm.nih.gov/sra/?term=PRJNA724821 and https://www.ncbi.nlm.nih.gov/sra/?term=PRJNA724811. Genbank accession number is CP075691 (https://submit.ncbi.nlm.nih.gov/subs/wgs/SUB9696180/overview).

## Author Contributions

NL and TP: data curation. YW: funding acquisition. NL, SC, ZY, JH, YT, and TP: investigation. NL, JH, and TP: methodology. YW: resources. NL: writing – original draft. NL and YW: writing – review and editing. All authors contributed to the article and approved the submitted version.

## Conflict of Interest

The authors declare that the research was conducted in the absence of any commercial or financial relationships that could be construed as a potential conflict of interest.

## Publisher’s Note

All claims expressed in this article are solely those of the authors and do not necessarily represent those of their affiliated organizations, or those of the publisher, the editors and the reviewers. Any product that may be evaluated in this article, or claim that may be made by its manufacturer, is not guaranteed or endorsed by the publisher.

## Footnotes

1
www.biomarker.com.cn

2
http://nrps.igs.umaryland.edu/

3
ftp://ftp.ebi.ac.uk/pub/software/clustalw2/2.1

4
www.ezbiocloud.net/tools/ani

5
http://ggdc.dsmz.de/ggdc.php#

## Supplementary Material

Supplementary Figure 1 *In vivo* antifungal activity of the fermentation broth of *Streptomyces* sp. 135 against *Sclerotinia sclerotiorum* in rape leaves.

Supplementary Figure 2 *In vivo* antifungal activity of the fermentation broth of *Streptomyces* sp. 135 against *Gaeumannomyces graminis* in wheat.

Supplementary Figure 3 *In vivo* antifungal activity of the fermentation broth of *Streptomyces* sp. 135 against *Phytophthora capsici* in pepper.

Supplementary Figure 4 Antifungal effects of X-14952B. **(A)** *Alternaria alternata*, **(B)** *Botrytis cinerea*, **(C)** *Fusarium graminearum*, **(D)** *Glomerella cingulata*, **(E)** *Gaeumannomyces graminis*, **(F)** *Phytophthora capsici*, and **(G)** *Sclerotinia sclerotiorum*.

Supplementary Figure 5 Antibacterial effects of X-14952B. **(A)** *Bacillus subtilis*, **(B)** *Xanthomonas oryzae*, **(C)** *Ralstonia solanacearum*, **(D)** *Pseudomonas syringae*, **(E)** *Erwinia carotovora*, **(F)** *Xanthomonas citri.* STR: Streptomycin, 1,000 mg/L.

Supplementary Figure 6 Maximum-likelihood phylogenetic tree Phylogenetic tree of *Streptomyces* based on almost complete 16S rRNA sequences (1,441 nucleotides) showing the relationship between strain 135 and representatives of some other related taxa. Numbers at nodes indicate percentages of 1,000 bootstrap resamplings. Bar, 0.005 substitutions per site.

Supplementary Figure 7 Maximum-parsimony phylogenetic tree Based on almost complete 16S rRNA sequences (1,441 nucleotides) showing the relationship between strain 135 and representatives of some other related taxa. Numbers at nodes indicate percentages of 1,000 bootstrap resamplings, only values above 50% are shown. Bar, 10 substitutions per site.

Supplementary Figure 8 Core-genome-based phylogenetic relationships of *Streptomyces* sp. 135 and other closely related species using the neighbor-joining algorithm. Numbers at nodes represent the percentage of 1,000 bootstrap resamples; only values > 50% are shown. Bar, 0.02 substitutions per site. GenBank assembly accession numbers of all genomes are consistent with Figure 1.

Supplementary Figure 9 Core-pan genome plot. The plot shows the increase of the pan-genome (blue) and the decrease of the core-genome (pink), and the new gene clusters of all 24 genomes. GenBank assembly accession numbers of all genomes are consistent with Figure 1. The corresponding numbers of genomes are list in below: (1) *Streptomyces* sp. 135 (2) *Streptomyces alboniger* ATCC 12461^T^ (3) *Streptomyces aureus* NRRL-B-1941^T^ (4) *Streptomyces aureus* NRRL-B-1941^T^ (5) *Streptomyces avermitilis* MA-4680^T^ (6) *Streptomyces badius* JCM4350^T^ (7) *Streptomyces bottropensis* ATCC 25435^T^ (8) *Streptomyces canus* DSM 40017^T^ (9) *Streptomyces flavovariabilis* NRRL B-16367^T^ (10) *Streptomyces formicae* 1H-GS9^T^ (11) *Streptomyces globisporus* TFH56^T^ (12) *Streptomyces griseorubiginosus* DSM 40469^T^ (13) *Streptomyces griseus* ATCC 13273^T^ (14) *Streptomyces huasconensis* HST28^T^ (15) *Streptomyces kanamyceticus* ATCC 12852^T^ (16) *Streptomyces microflavus* NBRC 13062^T^ (17) *Streptomyces mirabilis* P16B-1^T^ (18) *Streptomyces olivochromogenes* DSM 40451^T^ (19) *Streptomyces phaeoluteigriseus* DSM 41896^T^ (20) *Streptomyces pseudovenezuelae* DSM 40212^T^ (21) *Streptomyces resistomycificus* NRRL ISP-5133^T^ (22) *Streptomyces setonii* NRRL ATCC 13273^T^ (23) *Streptomyces stelliscabiei* NRRL B-24447^T^ (24) *Streptomyces variegatus* NRRL B-16380^T^.

Supplementary Figure 10 Functional annotation of predicted gene according to the COGs comparison of COGs of *Streptomyces* sp. 135 with phylogenetically related species of the genus *Streptomyces*.

Supplementary Figure 11 Petal diagram shows the pan-genome analysis of and four closely species in *Streptomyces*. Each strain is represented by a colored oval. The center is the number of orthologous coding sequences shared by all strains. Numbers in non-overlapping portions of each oval show the numbers of CDSs unique to each strain.

Supplementary Figure 12 Pan-genome tree of *Streptomyces* sp. 135 and other closely related species using the neighbor-joining algorithm. Numbers at nodes represent the percentage of 1,000 bootstrap resamples; only values > 50% are shown. Bar, 0.02 substitutions per site. GenBank assembly accession numbers of all genomes are consistent with Figure 1.

Supplementary Figure 13 Phylogenetic tree was built using maximum-likelihood method, Tamura–Nei model and 1,000 bootstrap replicates showing the relationship between strain 135 and representatives of some other related taxa. Only values above 50% are shown. Bar, 0.02 substitutions per site.

Supplementary Figure 14 ^1^H NMR (500 MHz, CD_3_OD) spectrum of X-14952B.

Supplementary Figure 15 ^13^C NMR (500 MHz, CD_3_OD) spectrum of X-14952B.

Supplementary Figure 16 HRESIMS spectrum of X-14952B(-).

Supplementary Figure 17 HRESIMS spectrum of X-14952B(+).

Supplementary Figure 18 Incomplete amino acid sequence alignment of AT, KR, DH, and ER domains in X-14952B biosynthesis, where the essential motifs are in red, and key catalytic residues are in blue. **(A)** AT domains. **(B)** KR domains, configurations were inferred by the Keatinge-Clay system. **(C)** DH domains. **(D)** ER domains.

Supplementary Table 1 Number of actinomycetes isolated from different areas.

Supplementary Table 2 General features of the genome of *Streptomyces* sp. 135.

Supplementary Table 3 Biosynthetic gene clusters of *Streptomyces* sp. 135.

Supplementary Table 4 ^1^H (500 MHz) and ^13^C (500 MHz) (CD_3_OD) NMR Data of X-14952B.
