# Supplementary material for: Antimicrobial Activity and Identification of the Biosynthetic Gene Cluster of X-14952B From Streptomyces sp. 135
Source: Front Microbiol. 2021 Aug 2;12:703093. doi: 10.3389/fmicb.2021.703093 (PMC8365161; doi:10.3389/fmicb.2021.703093)
Supplement: Supplementary Figure 1 — In vivo antifungal activity of the fermentation broth of Streptomyces sp. 135 against Sclerotinia sclerotiorum in rape leaves. [file Data_Sheet_1.pdf]

Supplementary Material

Antimicrobial Activity and Identification of the Biosynthetic Gene Cluster of X-14952B from *Streptomyces* sp. 135

Li Na[1,2], Chen Simin[3], Yan Zhiqiang[1], Han Jinhua[1], Ta Yongquan[1], Pu Taixun[1], Wang Yonghong[1]*

[1]Research and Development Center of Biorational Pesticides, Key Laboratory of Plant Protection Resources and Pest Management of Ministry of Education, Northwest A&F University, 22 Xinong Road, Yangling,Xianyang, Shaanxi 712100, China

[2]Institute Vegetable, Zhangye Academy of Agricultural Sciences, Seven kilometers of Zhang Su Highway, Zhangye, Gansu 734000, China

[3]College of Plant Protection, Northwest A&F University, 22 Xinong Road, Yangling, Xianyang, Shaanxi 712100 China

***Correspondence:**
Yonghong Wang
yhwang@nwafu.edu.cn

Email addresses:
Li Na: m18293605408@163.com
Chen Simin:simin_chen99@163.com
Yan Zhiqiang:yan0571@nwafu.edu.cn
Han Jinhua:hanjinhua95@163.com
Ta Yongquan: yongquanta@163.com
Pu Taixun:ptx675480306@163.com
Wang Yonghong:yhwang@nwafu.edu.cn

All media formulations used in this study

ISP2 media (g/L): Yeast Extract 4.0, Malt Extract 10.0, Dextrose 4.0 g, Agar 20.0, pH 7.2 ± 0.2.

ISP4 media (g/L): Soluble Starch 10.0, Dipotassium Phosphate 1.0, Magnesium Sulfate USP 1.0, Sodium Chloride 1.0, Ammonium Sulfate 2.0, Calcium Carbonate 2.0, Ferrous Sulfate 0.001, Manganous Chloride 0.001, Zinc Sulfate 0.001, Agar 20.0, pH 7.2 ± 0.2.

HVA media (g/L): Humic Acid 1.0, $CaCO_3$ 0.02, Na_2HPO_4 0.5, $FeSO_4 \cdot 7H_2O$ 0.01, KCl 1.7, $MgSO_4 \cdot 7H_2O$ 0.5, Thiamine 0.0005, Riboflavin 0.0005, Nicotinic Acid 0.0005, Calcium Pantothenate 0.0005, Vitamin B6 0.0005, Inositol 0.0005, p-Aminobenzoic Acid 0.0005, Biotin 0.00025, Agar 18, pH = 7.4 ± 0.2.

HSG media (g/L): Humic Acid 0.5, $CaCl_2$ 0.03, $FeSO_4 \cdot 7H_2O$ 0.001, $NiSO_4 \cdot 6H_2O$ 0.001, CHES 0.14, Ellanum 7.0, pH = 9.0 ± 0.2.

Millet media (g/L): Millet 10, Peptone 3, $CaCO_3$ 2, NaCl 2.5, pH 7.2 ± 0.2.

PDA media (g/L): Potato 200.0, D-glucose 20.0, Agar 15.0 -20.0, pH 7.2 ± 0.2.

TPA media (g/L): Trehalose 5.0, L- proline 1.0, $(NH_4)_2SO_4$ 1.0, CaCl 22.0, K_2HPO_4 1.0, $ZnSO_4.7H_2O$ 1.0, Thiamine 0.0005, Riboflavin 0.0005, Nicotinic Acid 0.0005, Pantothenic Acid 0.0005, Vitamin B6 0.0005, Inositol 0.0005, p-Aminobenzoic Acid 0.0005, Biotin 0.00025, Agar 20.0, pH = 7.2± 0.2.

Supplementary Table 1. Number of actinomycetes strains isolated from different geographic areas in the Qinghai-Tibet Plateau

Sampling site	No. of actinomycetes strain	Proportion (%)
Qinghai Lake	83	29. 75
Chaka	79	28. 32
Sichuan-Tibet Highway	17	6. 09
Danggula Mountains	15	5. 38
Amdo	14	5. 02
Quxu	11	3. 94
Lhasa	11	3. 94
Gangcha	9	323
Golmud	8	2. 87
Damxung	7	2. 51
Cambra	7	2. 51
Hoh Xil	6	215
Wudaoliang	5	1. 79
Naggu	4	1. 43
Tuotuo river	3	1. 08

Supplementary Table 2. General features of the genome of *Streptomyces* sp. 135

Sample	*Streptomyces* sp. 135
Length (bp)	8937250
Coding density (%)	88.25
N50 length (bp)	11883
N90 length (bp)	5985
Secondary metabolite biosynthetic gene coverage (%)	18.0
Average CDS length (bp)	955. 9
No. of protein-coding genes	8056
No. of tRNA genes	71
No. of reads	599074
No. of contigs	2
No. of scaffolds	2
No. of rRNA	18
No. of other ncRNAs	40
GC Content (%)	71. 44

Supplementary Table 3. Biosynthetic gene clusters of *Streptomyces* sp. 135

Region	Type	Cluster	Most similar known cluster	Similarity (%)
Region1. 1	CDPS	polyketide	Macrotermycins	7
Region1. 2	NRPS,betalactone T1PKS	t2pks	Murayaquinone	10
Region1. 3	lanthipeptide	lanthipeptide	Amf S	60
Region1. 4	T1PKS,NRPS-like	nrps-t1pks	Totopensamide	7
Region1. 5	T1PKS,NRPS	NRPS	GE81112	10
Region1. 6	T3PKS	t1pks	BE-14106	17
Region1. 7	NRPS	nrps-transatpks	Griseoviridin/viridogrisein	8
Region1. 8	Terpene	terpene	Isorenieratene	85
Region1. 9	terpene			
Region1. 10	T1PKS	t1pks	Concanamycin A	42
Region1. 11	ectoine	other	Ectoine	100
Region1. 12	NRPS,T1PKS			
Region1. 13	furan	other	Methylenomycin	14
Region1. 14	lanthipeptide			
Region1. 15	lassopeptide	ripp	Anantin C	75
Region1. 16	ladderane,NRPS	nrps-t2pks	Ishigamide	100
Region1. 17	T1PKS,butyrolactone	t1pks	Tetronasin	3
Region1. 18	terpene	terpene	Albaflavenone	100
Region1. 19	NRPS	other	Arginomycin	17
Region1. 20	siderophore	NRPS	Ficellomycin	3
Region1. 21	butyrolactone	t2pks-saccharide	Rabelomycin	6
Region1. 22	terpene,T3PKS	terpene	Pentalenolactone	58
Region1. 23	bacteriocin			
Region1. 24	terpene	terpene	Geosmin	100
Region1. 25	T2PKS	t2pks	Enterocin	85
Region1. 26	bacteriocin	t2pks	Asukamycin	3
Region1. 27	T1PKS	saccharide	Kanamycin	11
Region1. 28	terpene,NRPS-like	terpene	Hopene	92
Region1. 29	NRPS	NRPS	Enduracidin	18
Region1. 30	thiopeptide			

Region1. 31	lassopeptide	ripp	Citrulassin D	100
Region1. 32	betalactone,NRPS	NRPS	Friulimicin	30
Region1. 33	T3PKS,NRPS	NRPS	Feglymycin	73
Region1. 34	NRPS	saccharide	Acarviostatin	29
Region1. 35	NRPS	NRPS	Salinichelins	69
Region1. 36	NRPS,transAT-PKS	nrps-transatpks	Oxazolomycin	75
Region1. 37	NRPS,T1PKS	polyketide	Lavendiol	9%
Region2. 1	fused	saccharide	Tobramycin	

Supplementary Table 4. ^{1}H （500MHz） and ^{13}C （500MHz） (CD_3OD) NMR Data of X-14952B

Position	δC, type		δH，type		Position	δC, type		δH，type	
	actual	reported	actual	reported		actual	reported	actual	reported
1	174.58	173.7			22	32.59	32.8	1.74	1.53
2	45.87	43.6	Ha:2.51<br>Hb:2.45	2.57<br>2.67	23	79.74	77.0	3.67	3.55
3	99.64	94.3			24	58.59	55.4	2.62	2.67
4	36.34	35.3	2.11	2.1-2.2	25	218.43	217.5		
5	118.57	117.0	5.40	5.50	26	40.29	37.4	2.46	2.50
6	133.29	133.7			27	11.82	7.6	1.24	1.00
7	81.89	80.3	4.51	4.46	28	18.67	17.4	0.90	0.88
8	135.40	135.4			29	7.83	5.7	0.89	0.85
9	131.44	129.7	5.46	5.44	30	17.34	16.0	0.85	0.80
10	28.95	27.3	Ha:2.11<br>Hb:1.82	2.10<br>1.8	31	11.34	12.9	0.87	0.83
11	27.6	26.2	1.31	1.2-1.3	32	23.36	22.9	1.593	1.58
12	36.30	35.5	1.56	1.68	33	12.30	12.0	0.86	0.84
13	95.81	82.6	3.93	3.98	34	11.11	11.0	1.39	1.39
14	135.14	134.8	5.53	5.56	35	20.69	19.2	1.44	1.49
15	134.36	134.6	5.21	5.24	1\`	118.57	98.4	4.55	4.57
16	45.4	42.3	2.11	2.13	2\`	39.02	37.0	2.3<br>1.63	2.28<br>1.68
17	78.49	78.2	3.40	3.26	3\`’	75.80	75.2	4.67	4.65
18	36.0	34.9	1.95	1.97	4\`	75.52	74.4	3.20	3.30
19	83.85	82.2	4.76	4.85	5\`	73.69	72.2	3.25	3.30
20	33.59	33.5	1.73	1.75	6\`’	19.56	17.7	1.28	1.32
21	38.78	37.2	Ha:1.17<br>Hb:0.93	1.20<br>0.95	3\`-$OCONH_2$	159.67	157.7		

Note: Compared with reported data from Tian[1]

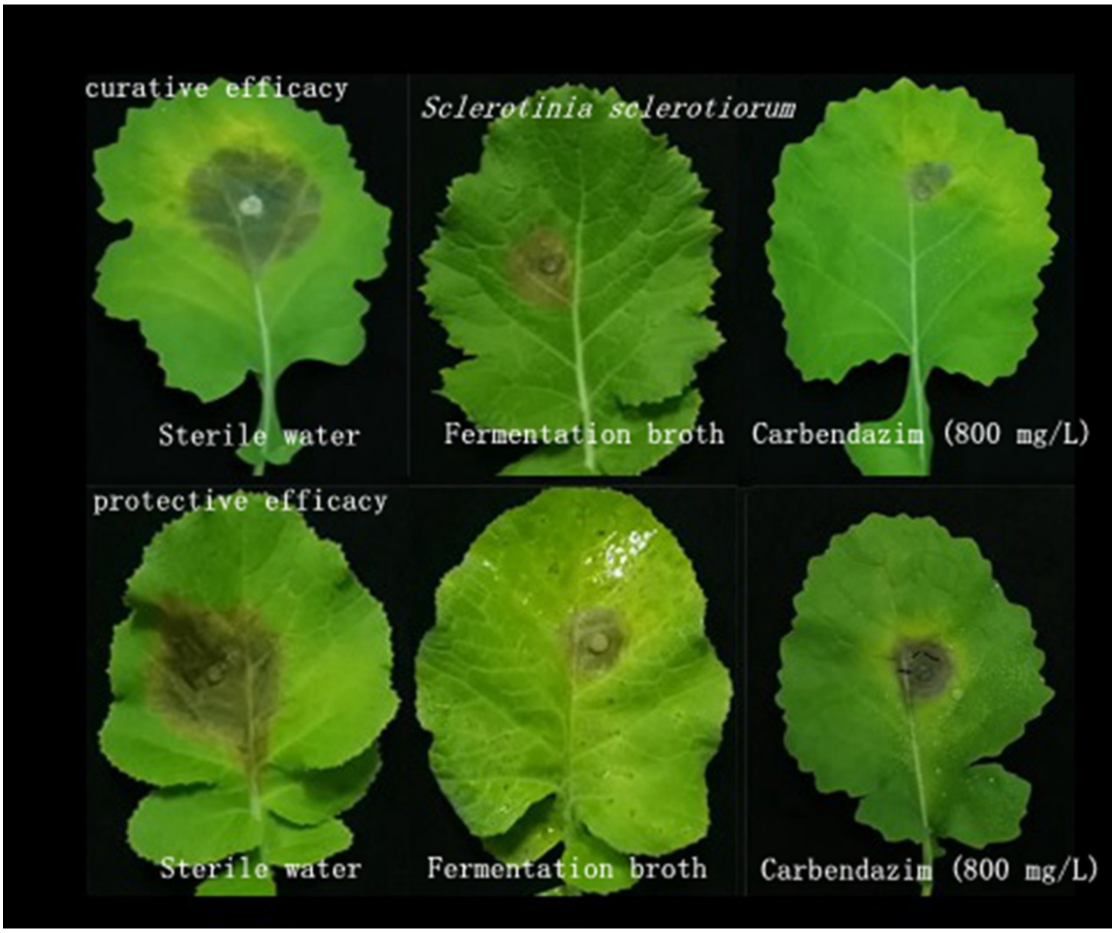

Supplementary Figure 1. *In vivo* antifungal activity of the fermentation broth of *Streptomyces* sp.135 against *Sclerotinia sclerotiorum* in rape leaves.

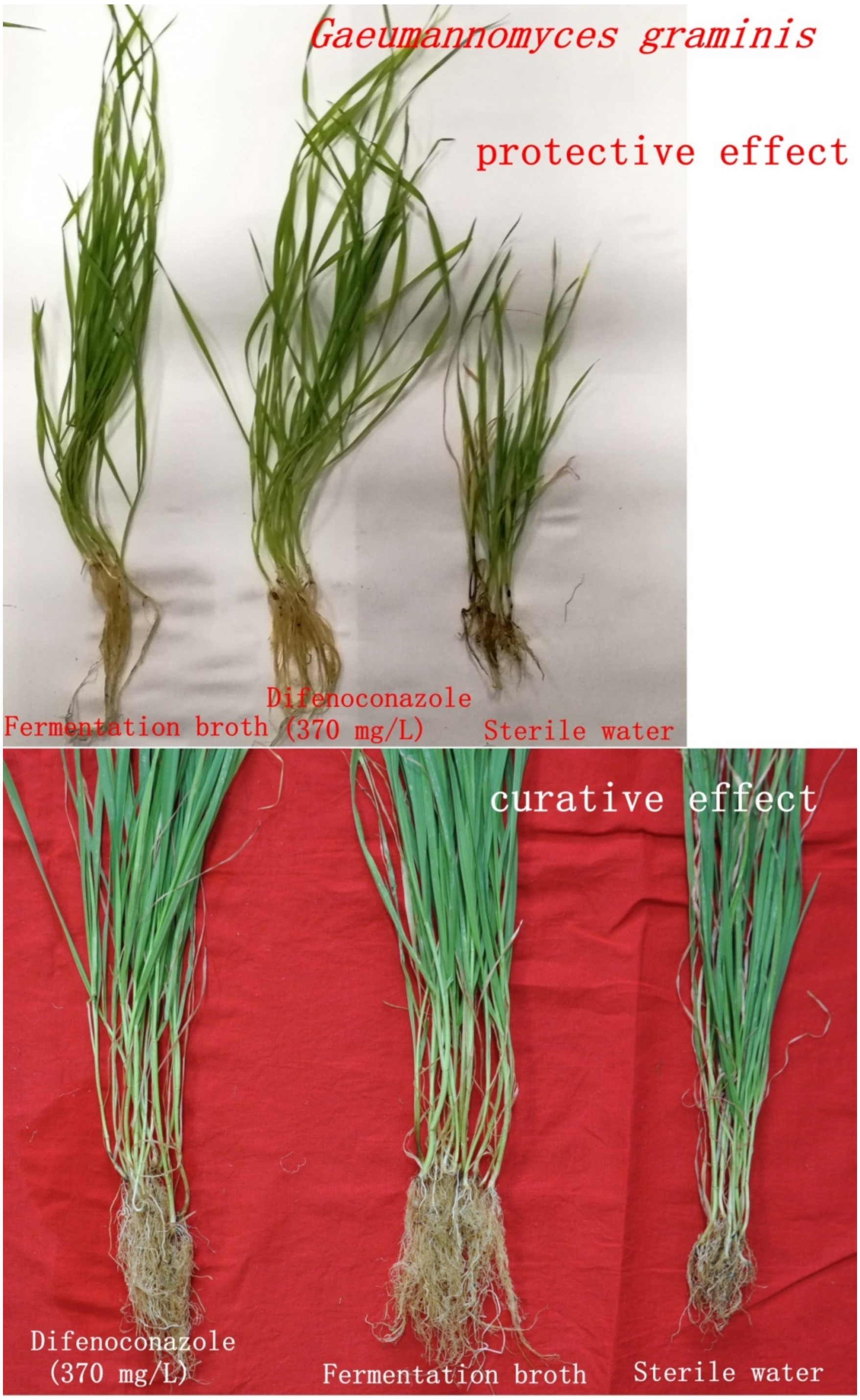

Supplementary Figure 2. *In vivo* antifungal activity of the fermentation broth of *Streptomyces* sp.135 against *Gaeumannomyces graminis* in wheat.

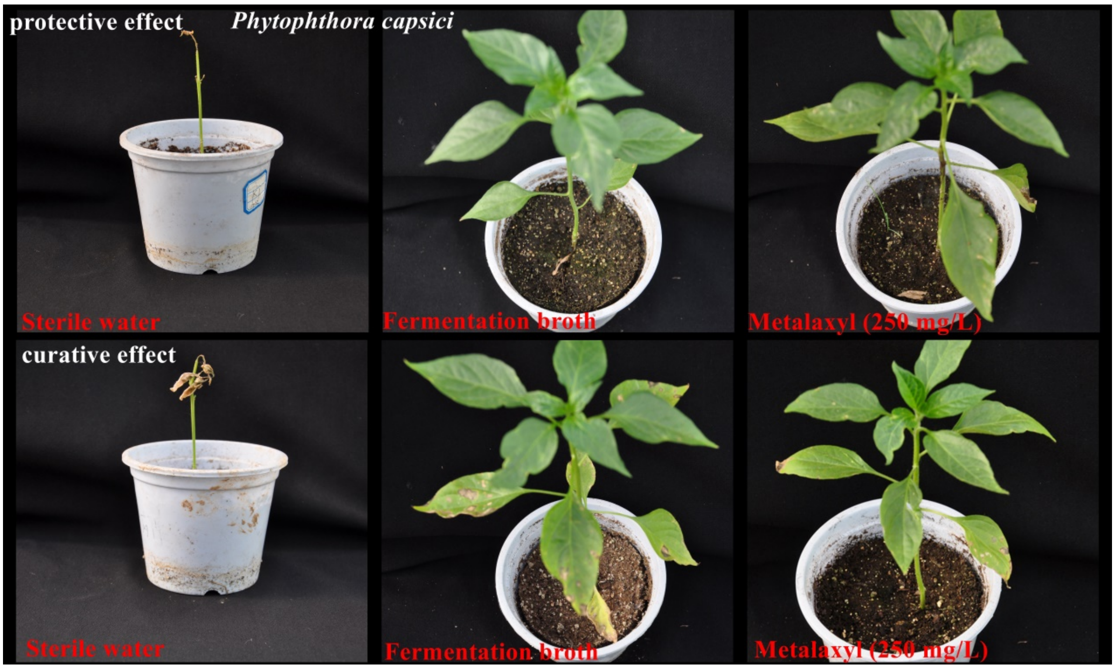

Supplementary Figure 3. *In vivo* antifungal activity of the fermentation broth of *Streptomyces* sp.135 against *Phytophthora capsici* in pepper.

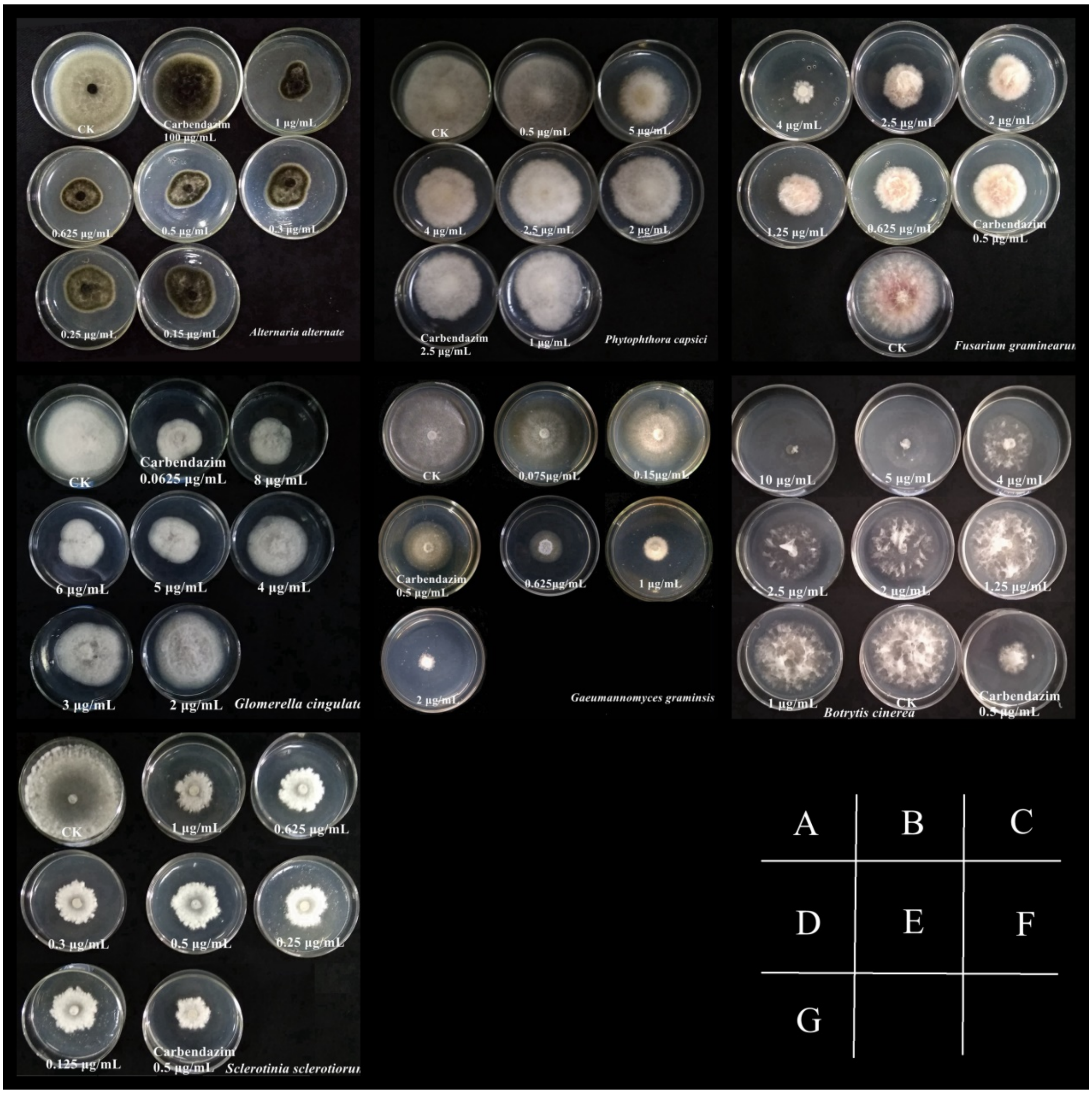

Supplementary Figure 4. Antifungal effects of X-14952B. A: *Alternaria alternata,* B: *Botrytis cinerea,* C: *Fusarium graminearum,* D: *Glomerella cingulata,* E: *Gaeumannomyces graminis,* F: *Phytophthora capsici,* G: *Sclerotinia sclerotiorum*

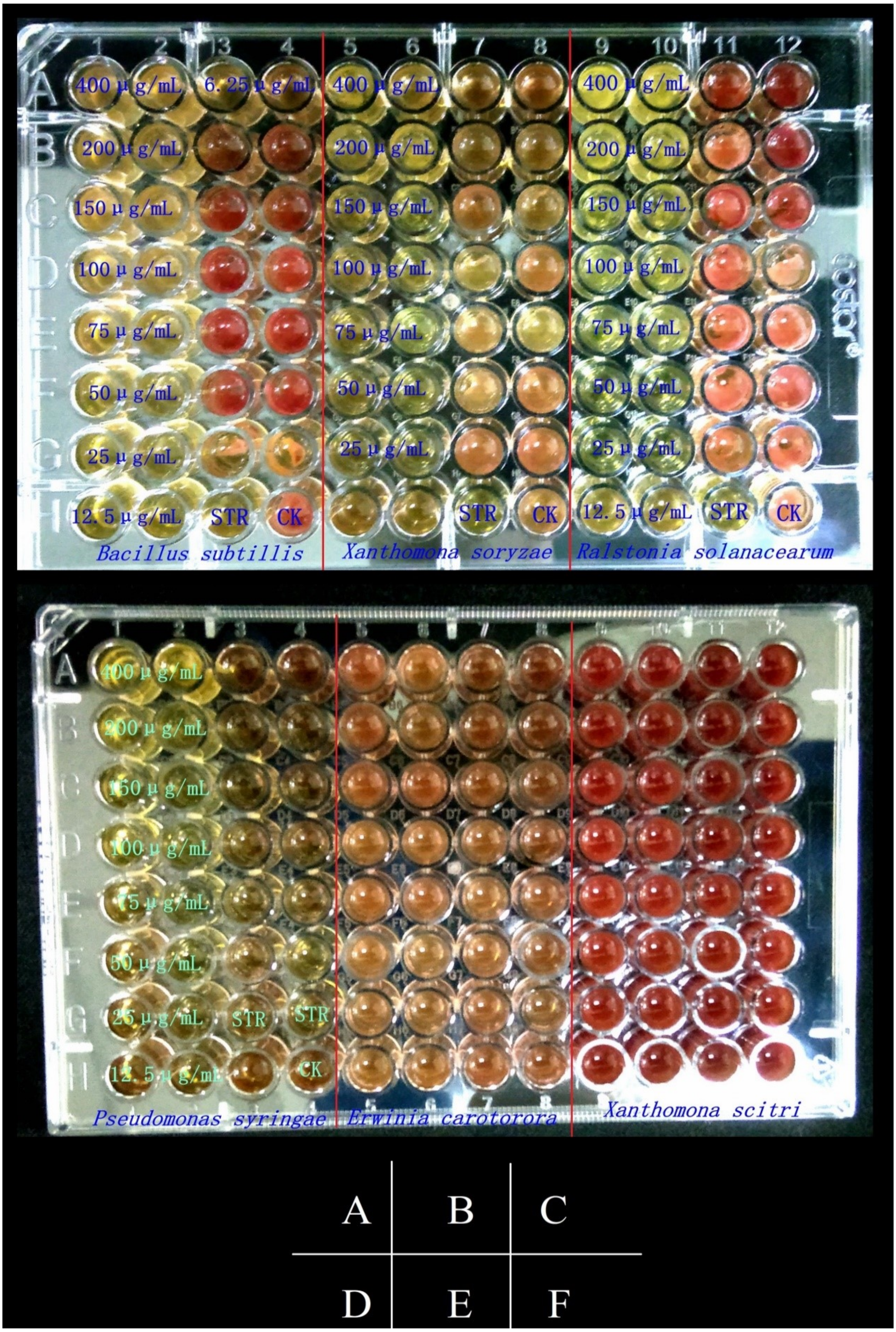

Supplementary Figure 5. Antibacterial effects of X-14952B. A: *Bacillus subtilis,* B: *Xanthomonas oryzae,* C: *Ralstonia solanacearum,* D: *Pseudomonas syringae,* E: *Erwinia carotorora,* F: *Xanthomonas citri.* STR: Streptomycin,1000mg/L

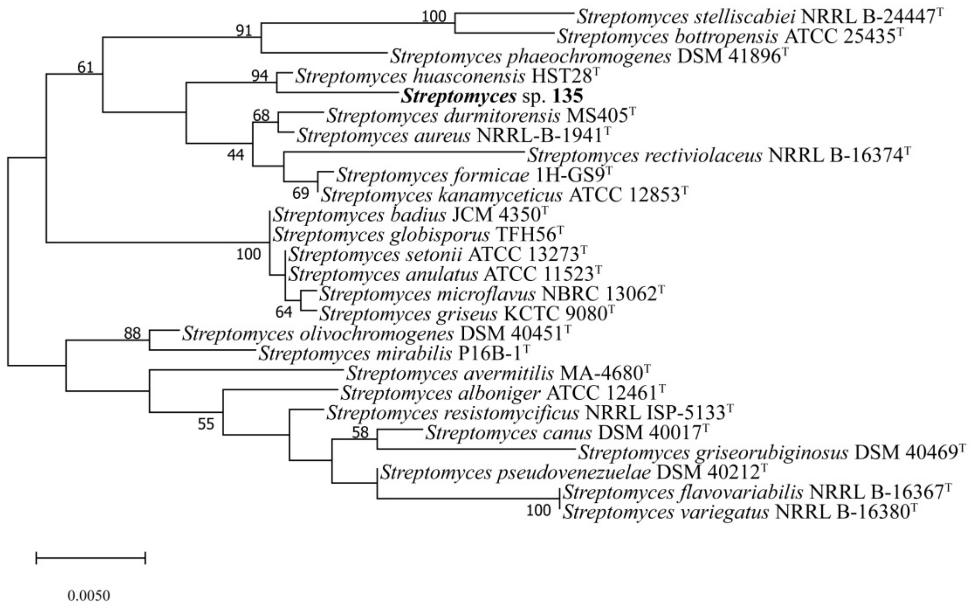

Supplementary Figure 6. Maximum-likelihood phylogenetic tree Phylogenetic tree of *Streptomyces* based on almost complete 16S rRNA sequences (1441 nucleotides) showing the relationship between strain 135 and representatives of some other related taxa. Numbers at nodes indicate percentages of 1000 bootstrap resamplings. Bar, 0.005 substitutions per site.

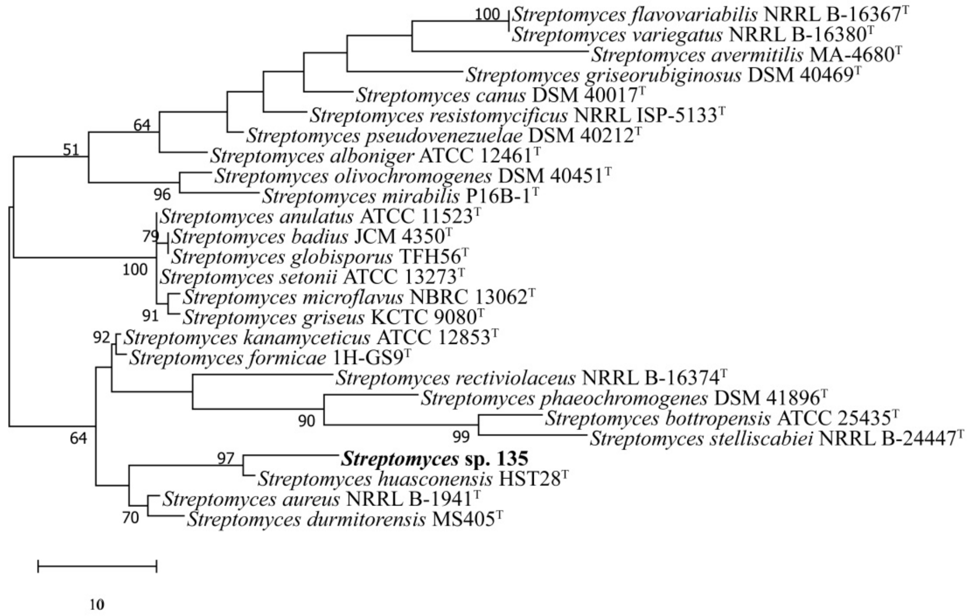

Supplementary Figure 7. Maximum-parsimony phylogenetic tree Based on almost complete 16S rRNA sequences (1441 nucleotides) showing the relationship between strain 135 and representatives of some other related taxa. Numbers at nodes indicate percentages of 1000 bootstrap resamplings, only values above 50% are shown. Bar, 10 substitutions per site.

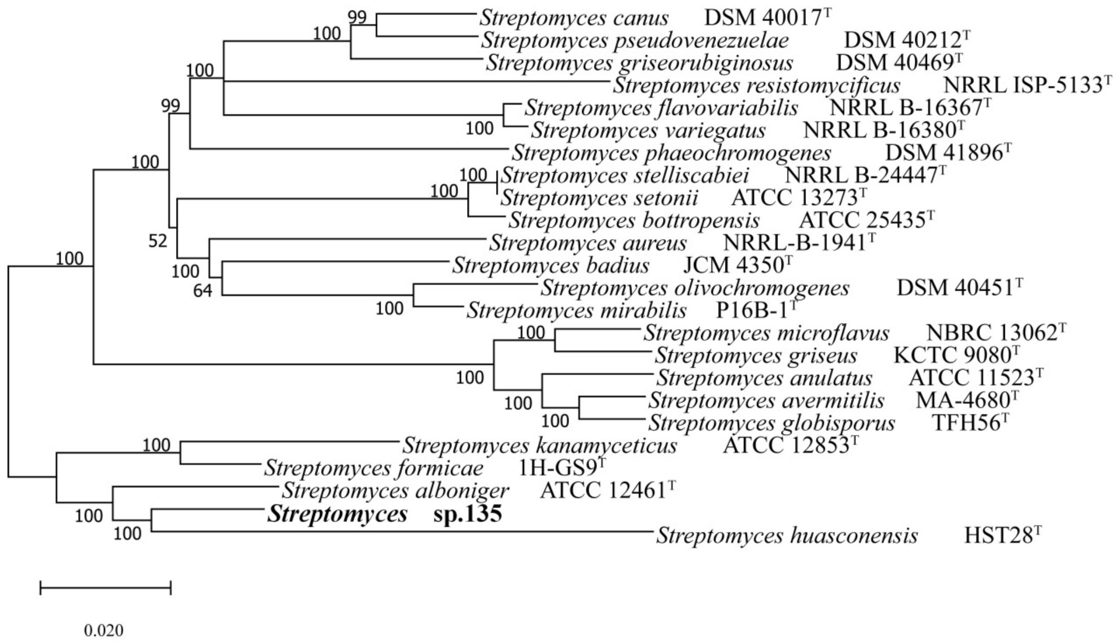

Supplementary Figure 8. Core-genome-based phylogenetic relationships of *Streptomyces* sp.135 and other closely related species using the neighbor-joining algorithm. Numbers at nodes represent the percentage of 1000 bootstrap resamples; only values >50% are shown. Bar, 0.02 substitutions per site. GenBank assembly accession numbers of all genomes are consistent with Figure 1.

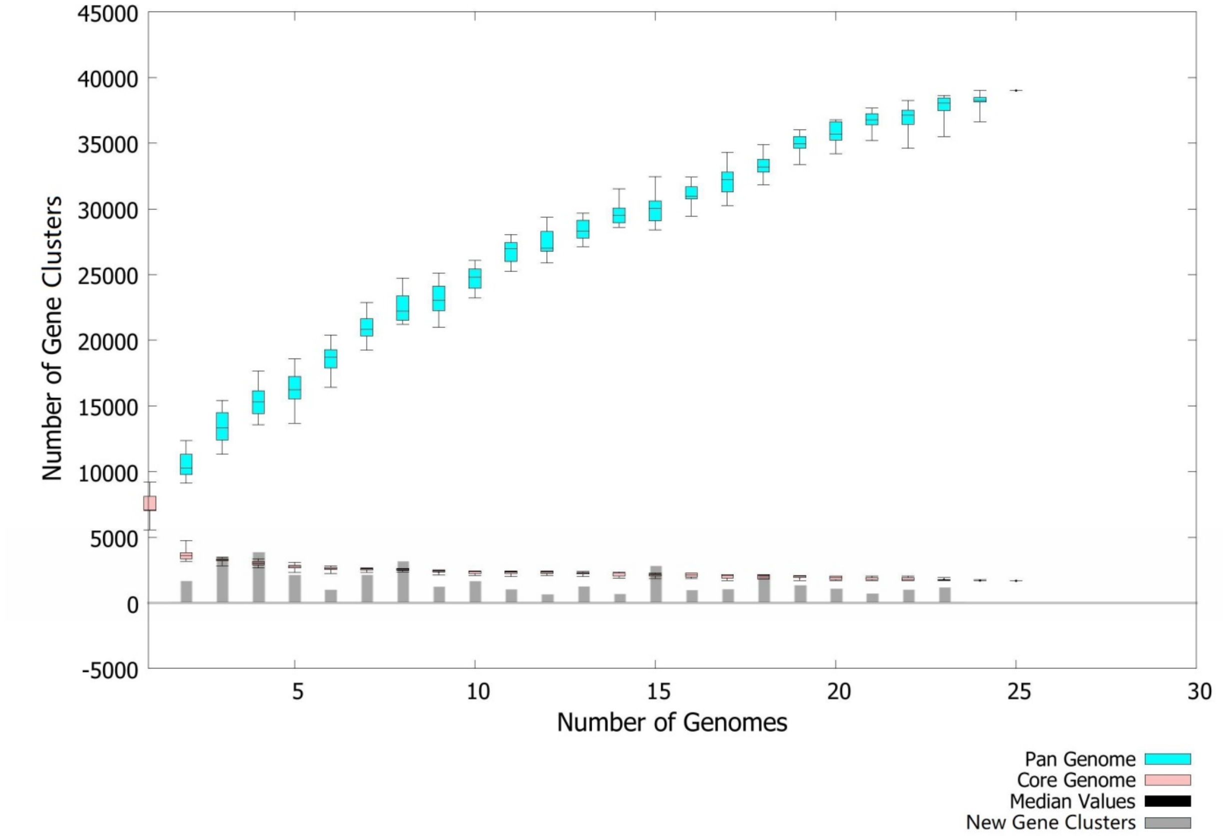

Supplementary Figure 9. Core-pan genome plot. The plot shows the increase of the pan-genome (blue) and the decrease of the core-genome (pink), and the new gene clusters of all 24 genomes. GenBank assembly accession numbers of all genomes are consistent with Figure 1. The corresponding numbers of genomes are list in below:

1. *Streptomyces* sp.135
2. *Streptomyces alboniger* ATCC 12461 ^{T}
3. *Streptomyces aureus* NRRL-B-1941 ^{T}
4. *Streptomyces aureus* NRRL-B-1941 ^{T}
5. *Streptomyces avermitilis* MA-4680 ^{T}
6. *Streptomyces badius* JCM4350 ^{T}
7. *Streptomyces bottropensis* ATCC 25435 ^{T}
8. *Streptomyces canus* DSM 40017 ^{T}
9. *Streptomyces flavovariabilis* NRRL B-16367 ^{T}
10. *Streptomyces formicae* 1H-GS9 ^{T}
11. *Streptomyces globisporus* TFH56 ^{T}
12. *Streptomyces griseorubiginosus* DSM 40469 ^{T}
13. *Streptomyces griseus* ATCC 13273 ^{T}
14. *Streptomyces huasconensis* HST28 ^{T}
15. *Streptomyces kanamyceticus* ATCC 12852 ^{T}
16. *Streptomyces microflavus* NBRC 13062 ^{T}
17. *Streptomyces mirabilis* P16B-1 ^{T}
18. *Streptomyces olivochromogenes* DSM 40451 ^{T}
19. *Streptomyces phaeoluteigriseus* DSM 41896 ^{T}
20. *Streptomyces pseudovenezuelae* DSM 40212 ^{T}
21. *Streptomyces resistomycificus* NRRL ISP-5133 ^{T}

22. *Streptomyces setonii* NRRL ATCC 13273 T
23. *Streptomyces stelliscabiei* NRRL B-24447 T
24. *Streptomyces variegatus* NRRL B-16380 T

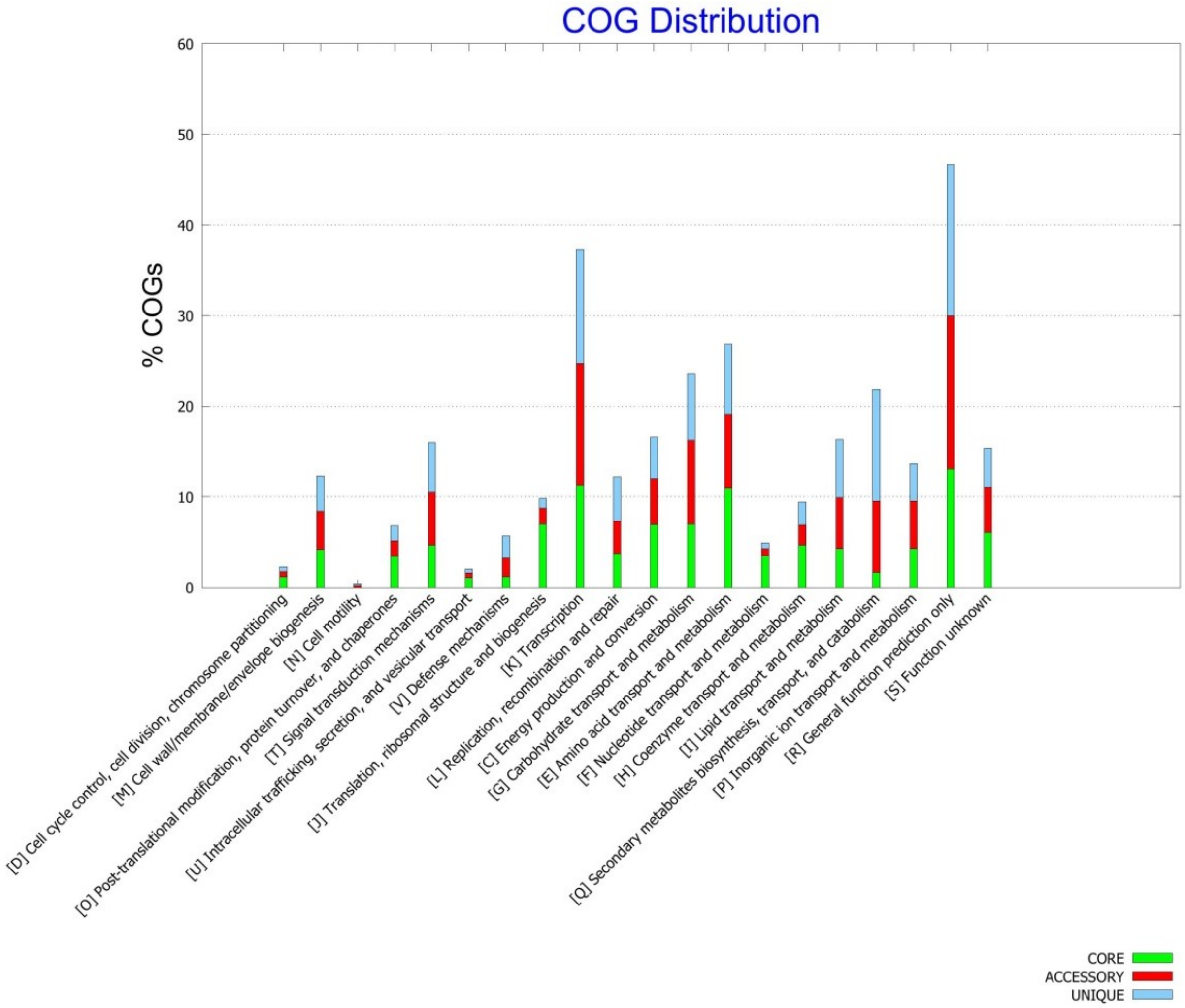

Supplementary Figure 10. Functional annotation of predicted gene according to the COGs comparison of COGs of *Streptomyces* sp.135 with phylogenetically related species of the genus *Streptomyces.*

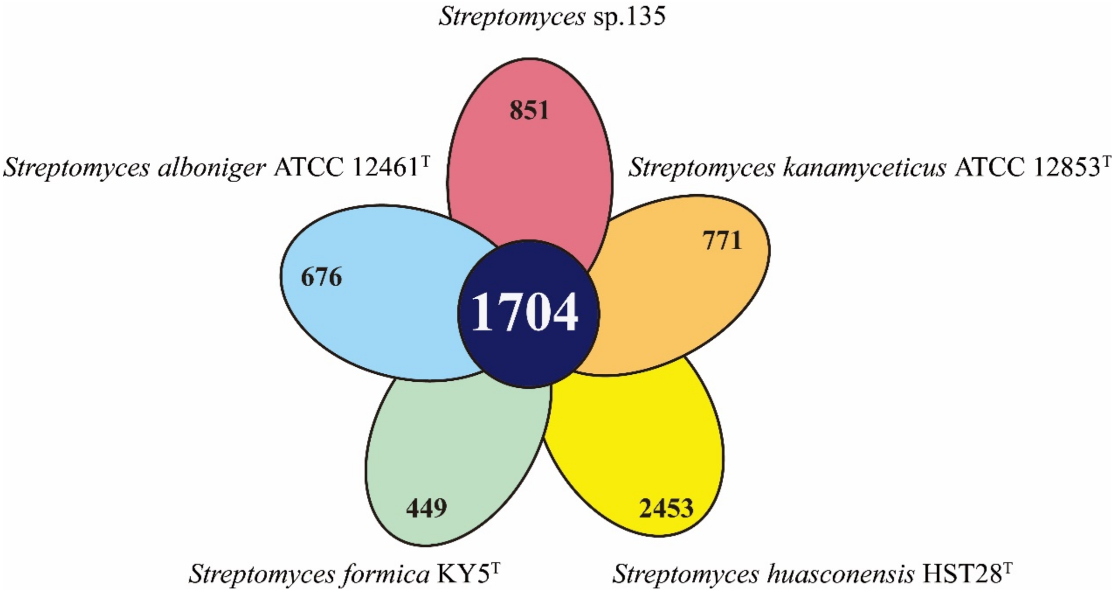

Supplementary Figure 11. Petal diagram shows the pan-genome analysis of and four closely species in *Streptomyces*. Each strain is represented by a colored oval. The center is the number of orthologous coding sequences shared by all strains. Numbers in non-overlapping portions of each oval show the numbers of CDSs unique to each strain.

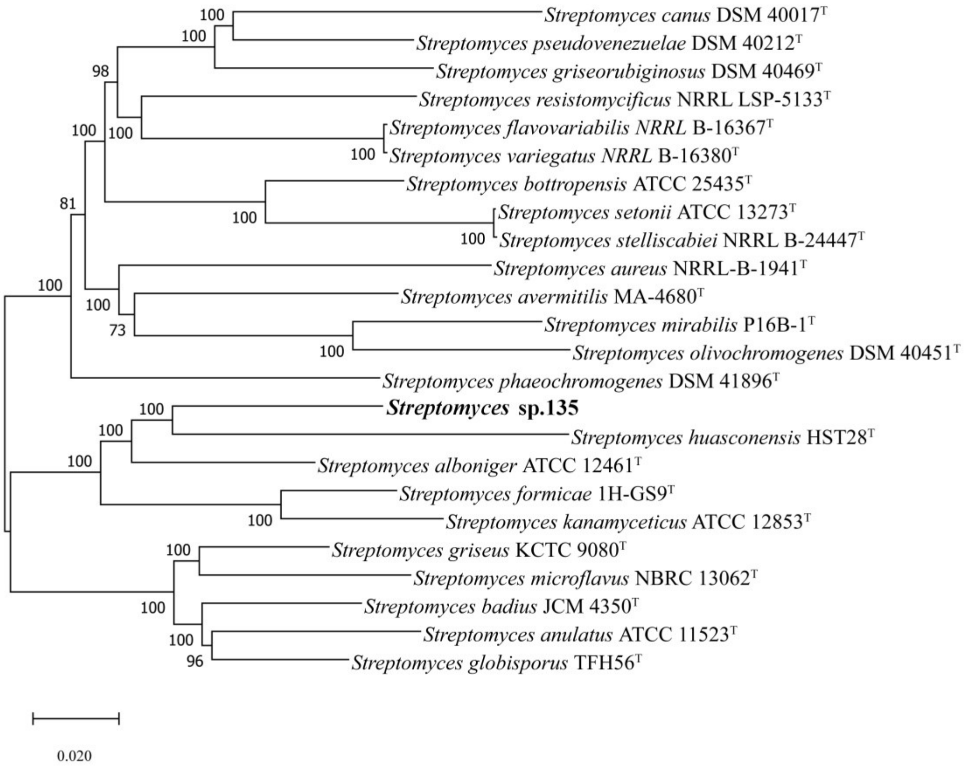

Supplementary Figure 12. Pan-genome tree of *Streptomyces* sp.135 and other closely related species using the neighbor-joining algorithm. Numbers at nodes represent the percentage of 1000 bootstrap resamples; only values >50% are shown. Bar, 0.02 substitutions per site. GenBank assembly accession numbers of all genomes are consistent with Figure 1.

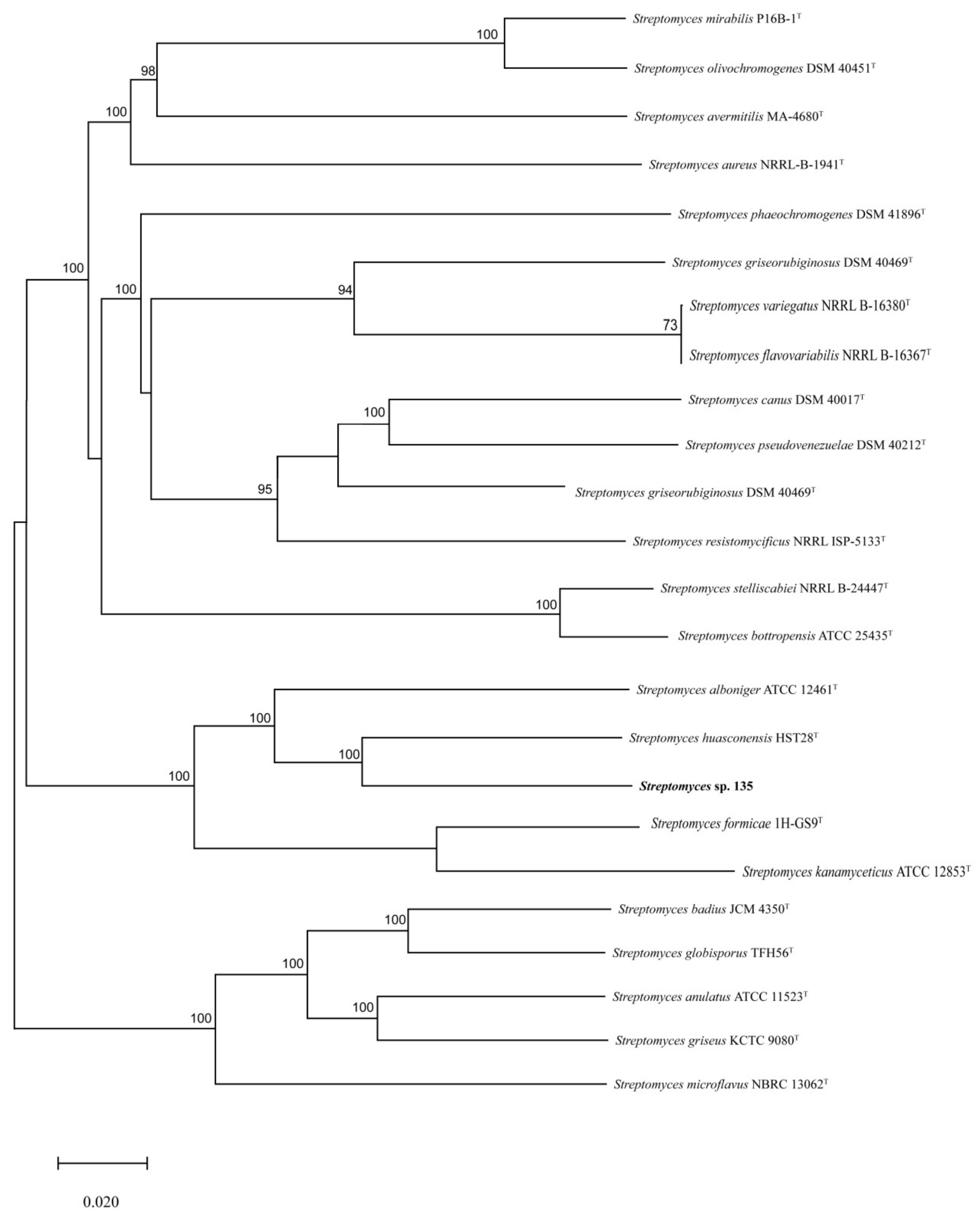

Supplementary Figure 13. Phylogenetic tree was built using maximum-likelihood method, Tamura–Nei model and 1,000 bootstrap replicates showing the relationship between strain 135 and representatives of some other related taxa. Only values above 50% are shown. Bar, 0.02 substitutions per site.

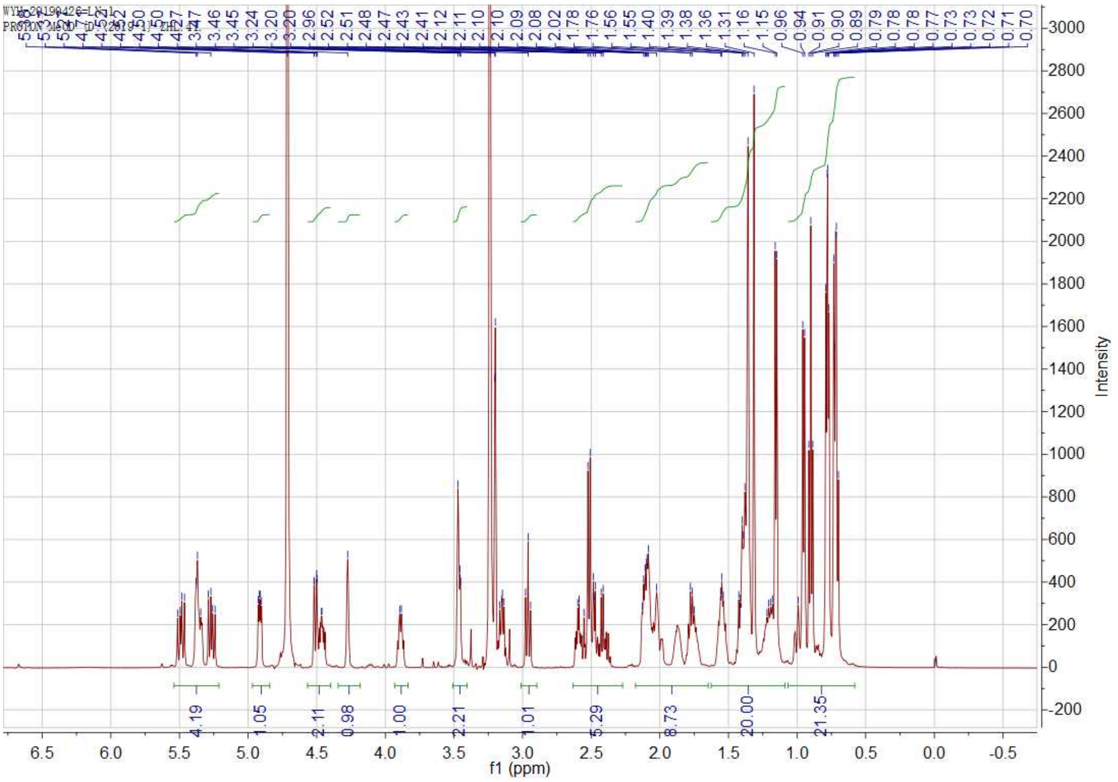

Supplementary Figure 14. ^{1}H NMR (500 MHz, CD_3OD) spectrum of X-14952B

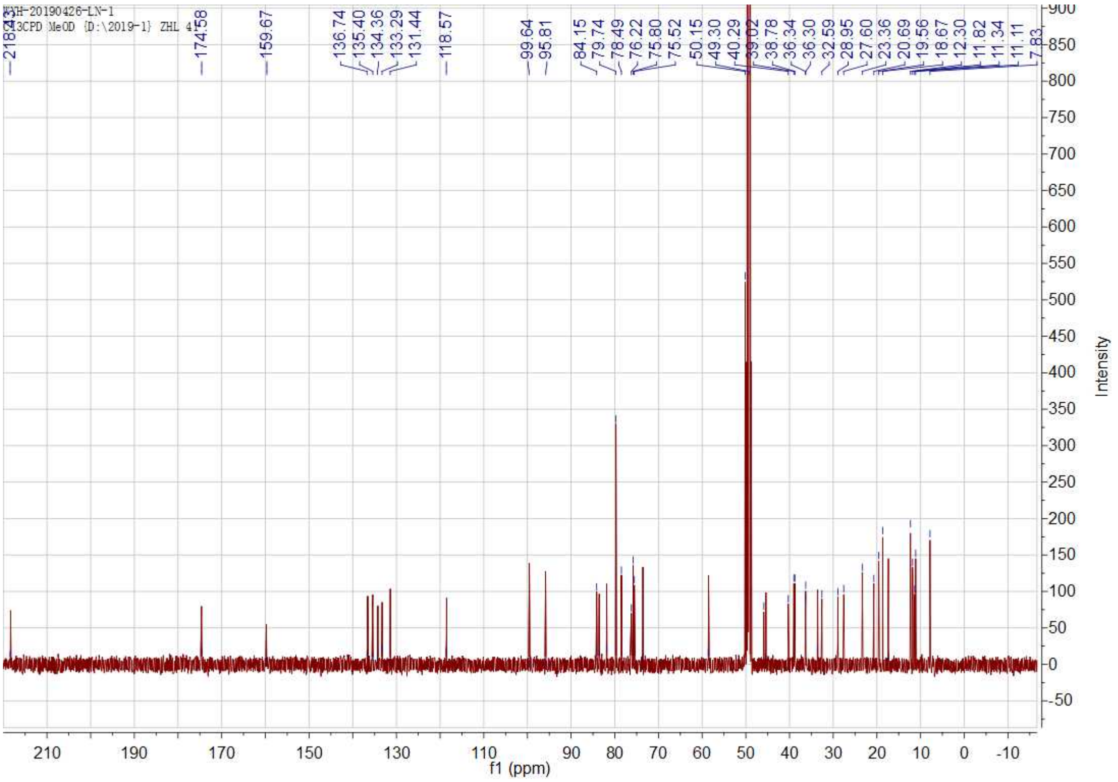

Supplementary Figure 15. ^{13}C NMR (500 MHz, CD_3OD) spectrum of X-14952B

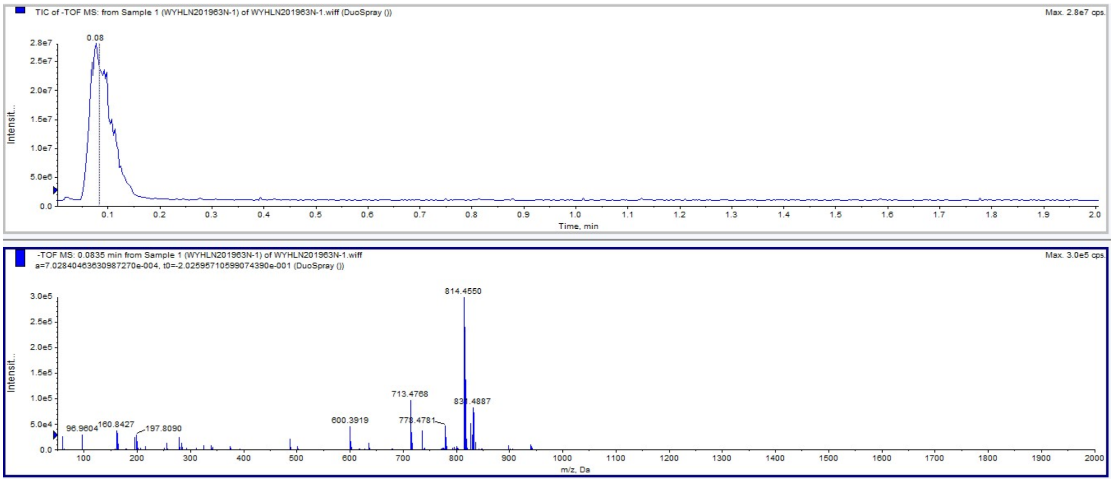

Supplementary Figure 16. HRESIMS spectrum of X-14952B（-）

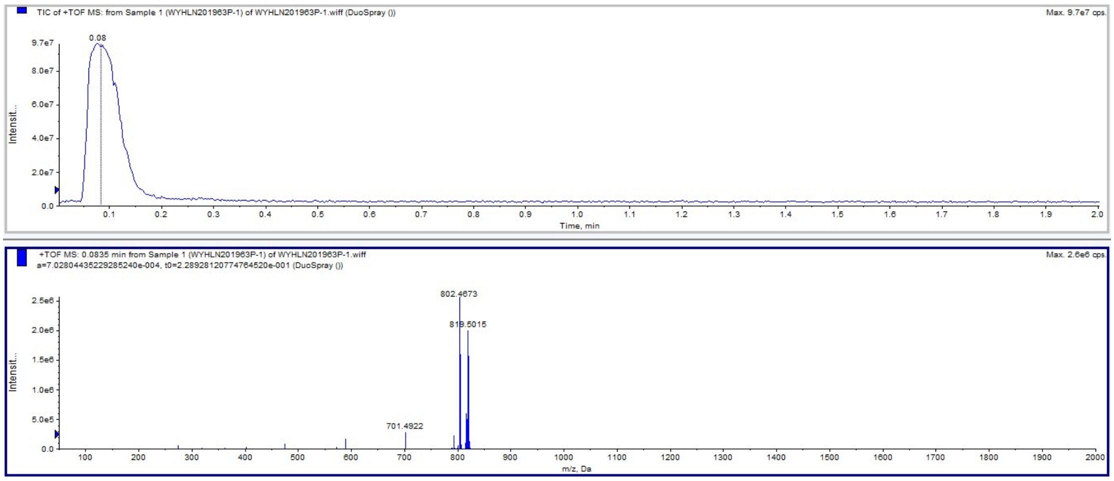

Supplementary Figure 17. HRESIMS spectrum of X-14952B（+）

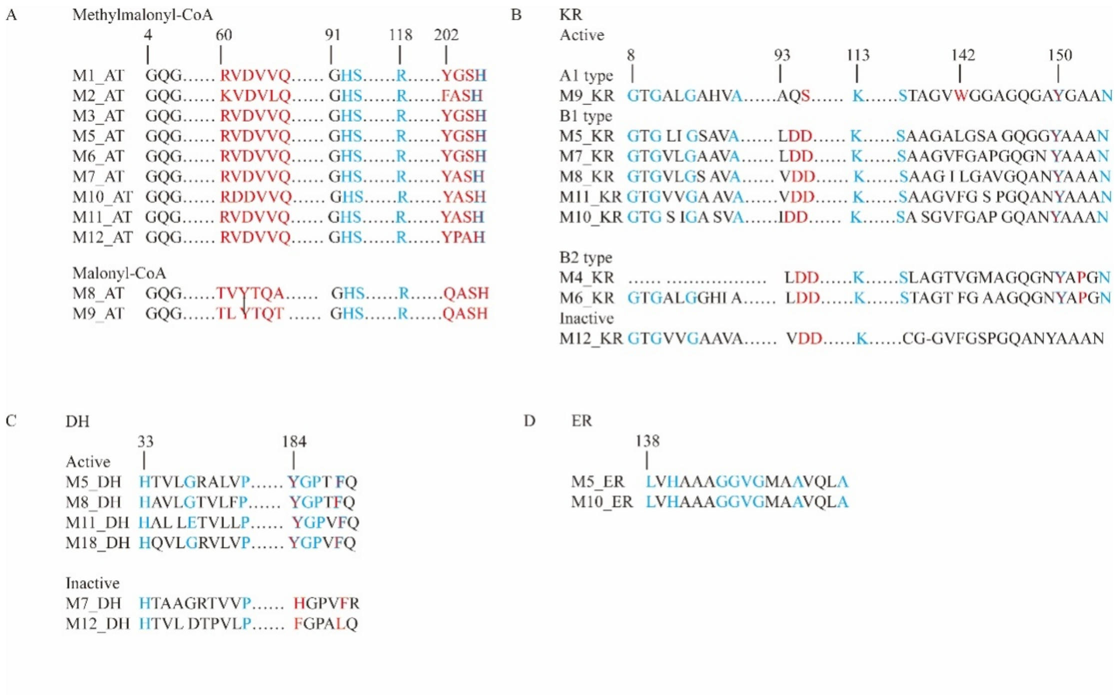

Supplementary Figure 18. Incomplete amino acid sequence alignment of AT, KR, DH, and ER domains in X-14952B biosynthesis, where the essential motifs are in red, and key catalytic residues are in blue. (**A**) AT domains. (**B**) KR domains, configurations were inferred by the Keatinge-Clay system. (**C**) DH domains. (**D**) ER domains.

Reference

1. Tian, H.; Shafi, J.; Ji, M.; Bi, Y.; Yu, Z. Antimicrobial Metabolites from Streptomyces Sp. SN0280. *J. Nat. Prod.***2017**, *80*, 1015–1019, doi:10.1021/acs.jnatprod.6b01016.
