# Supplementary figures and images for: Antimicrobial Activity and Identification of the Biosynthetic Gene Cluster of X-14952B From Streptomyces sp. 135
Source: Front Microbiol. 2021 Aug 2;12:703093. doi: 10.3389/fmicb.2021.703093 (PMC8365161; doi:10.3389/fmicb.2021.703093)

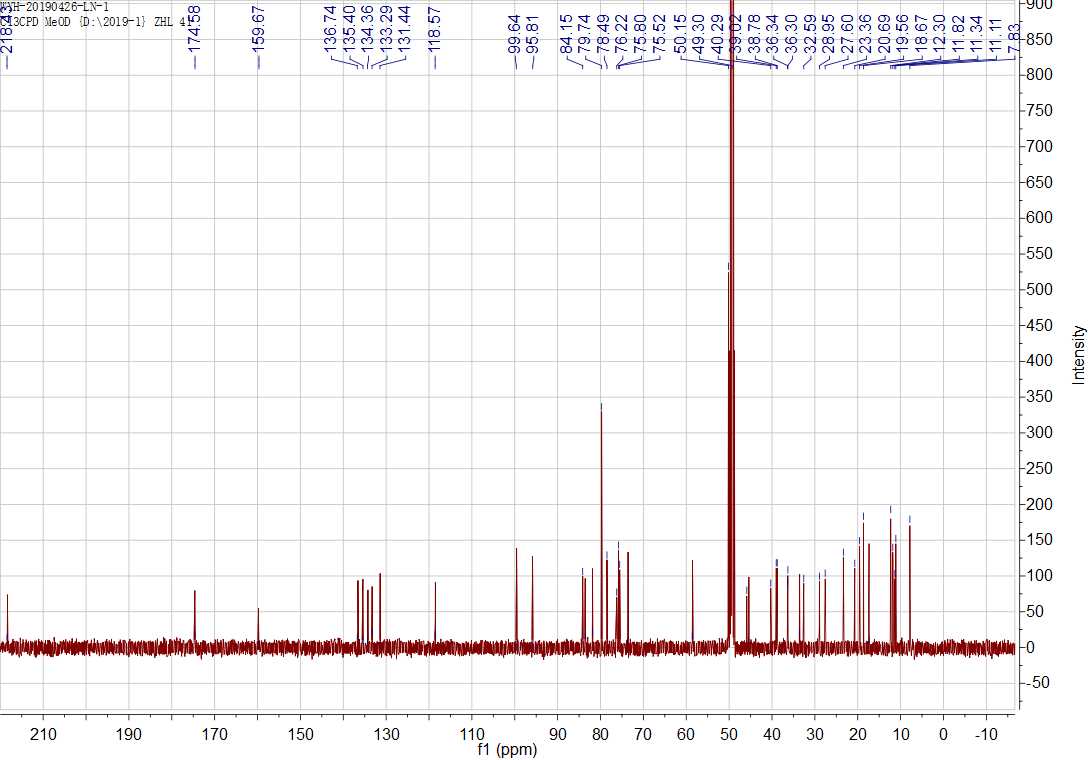

Supplement: Supplementary file 2 [file Data_Sheet_1.ZIP › figures1/13C NMR (500 MHz, CD3OD) spectrum of X-14952B.jpeg]

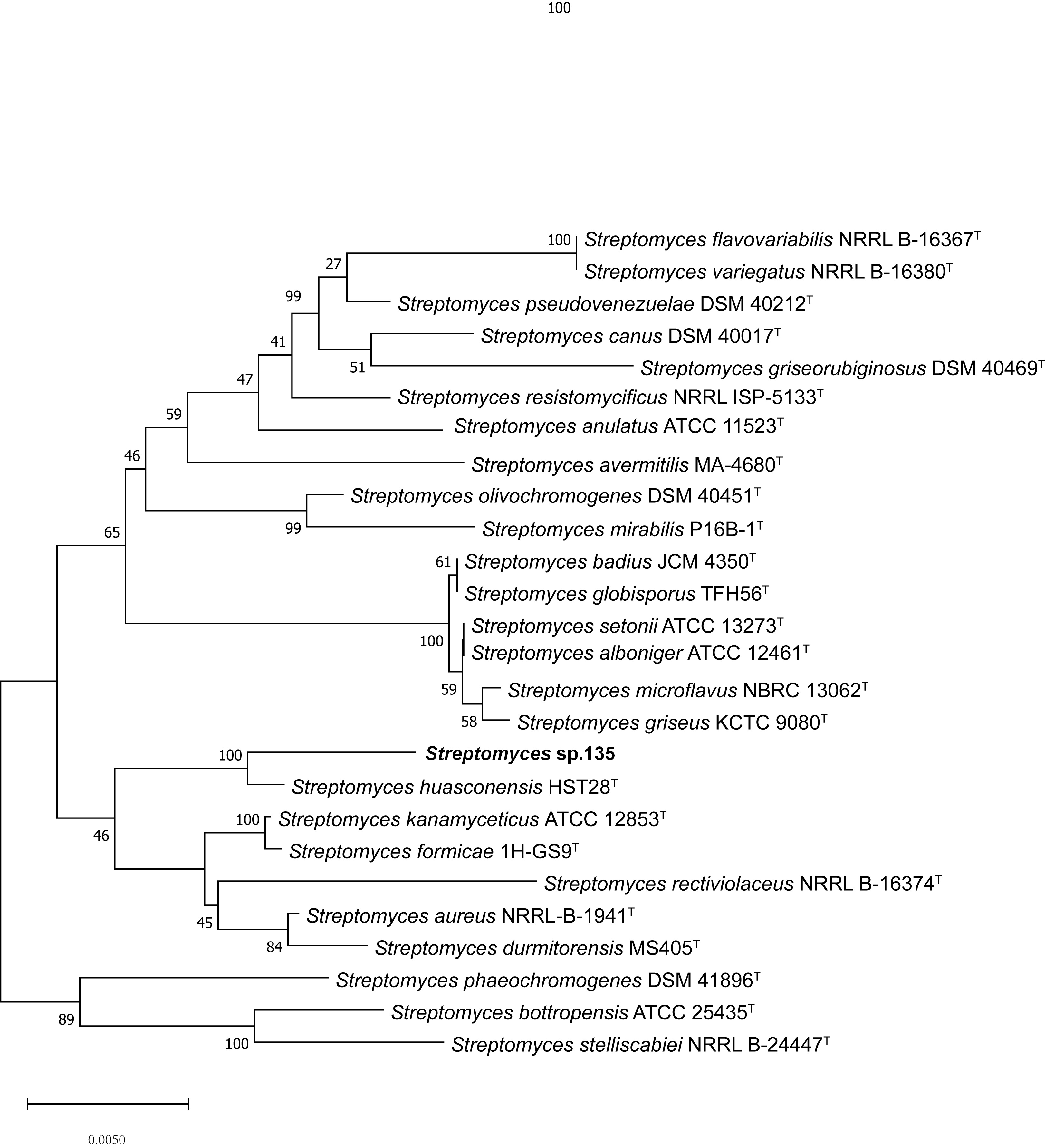

Supplement: Supplementary file 2 [file Data_Sheet_1.ZIP › figures1/16s NJ tree normal.jpg]

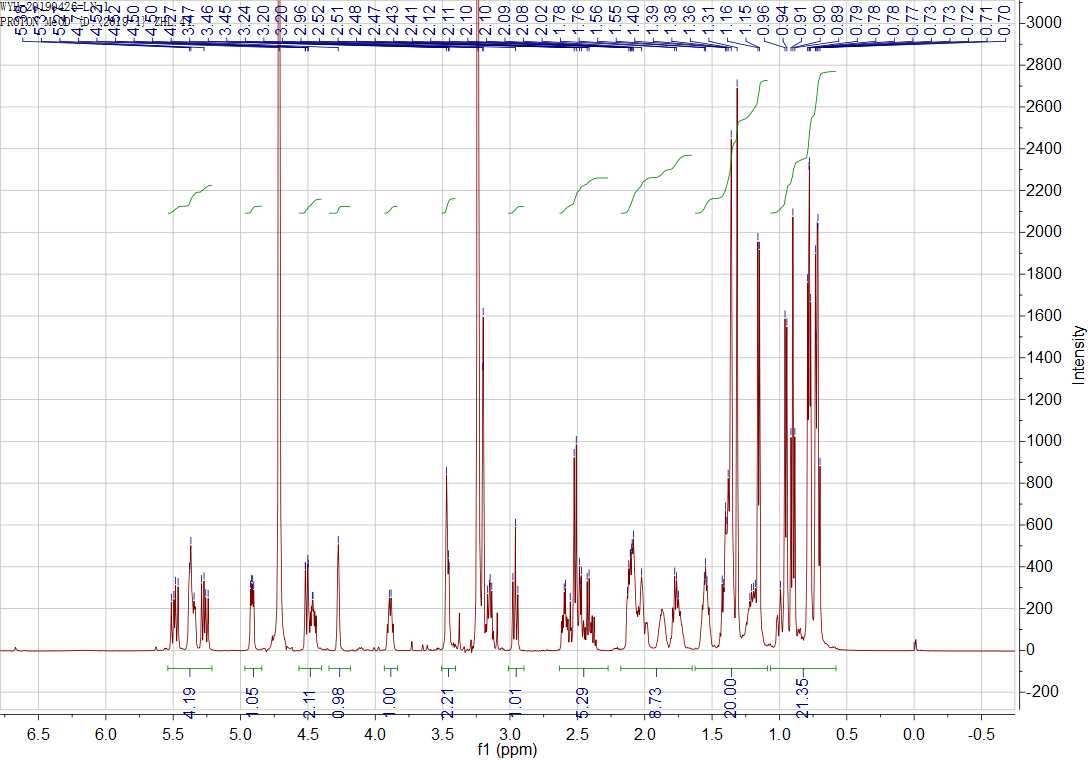

Supplement: Supplementary file 2 [file Data_Sheet_1.ZIP › figures1/1H NMR (500 MHz, CD3OD) spectrum of X-14952B.jpeg]

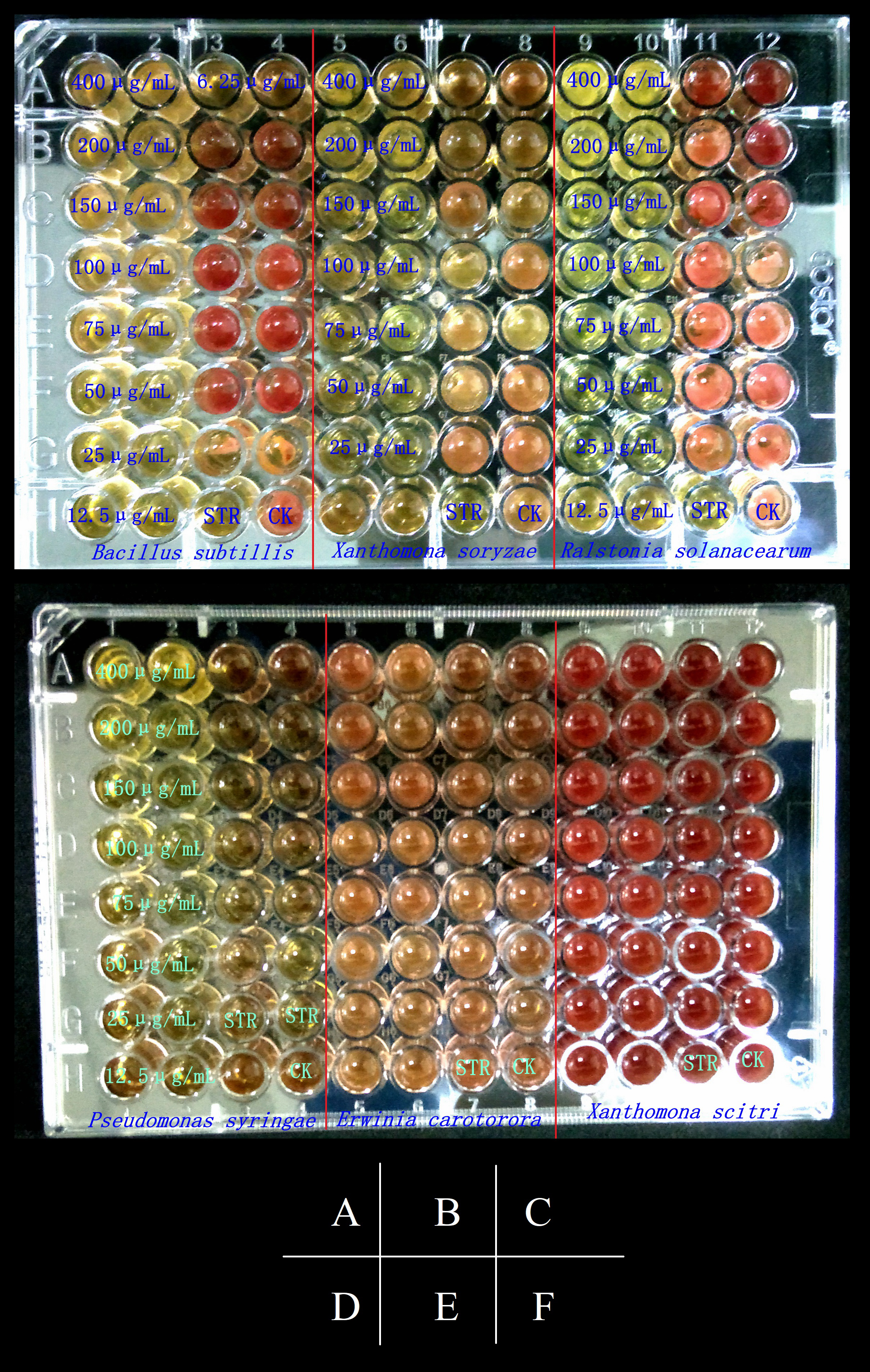

Supplement: Supplementary file 2 [file Data_Sheet_1.ZIP › figures1/Antibacterial effects of X-14952B.jpg]

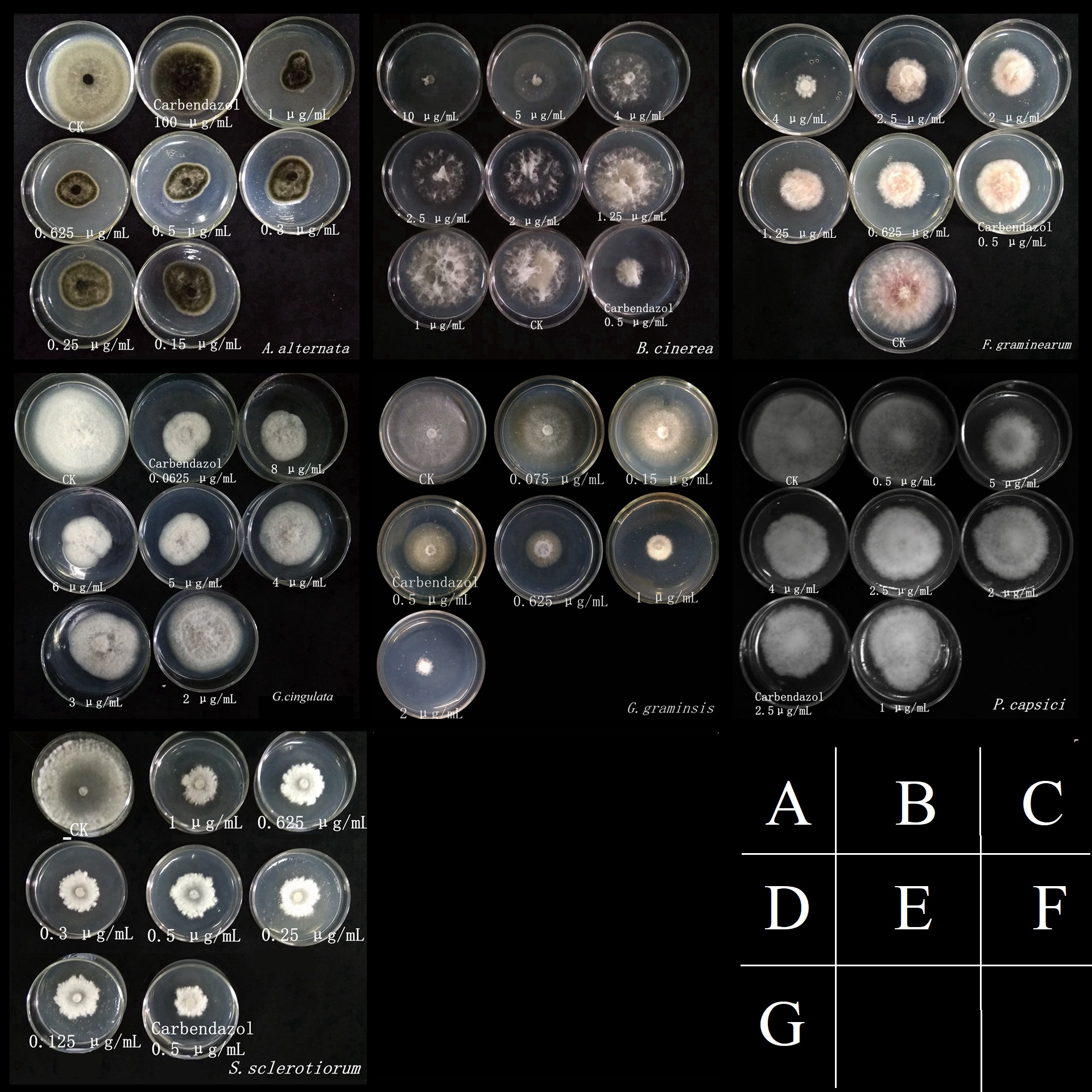

Supplement: Supplementary file 2 [file Data_Sheet_1.ZIP › figures1/Antifungal effects of X-14952B.jpg]

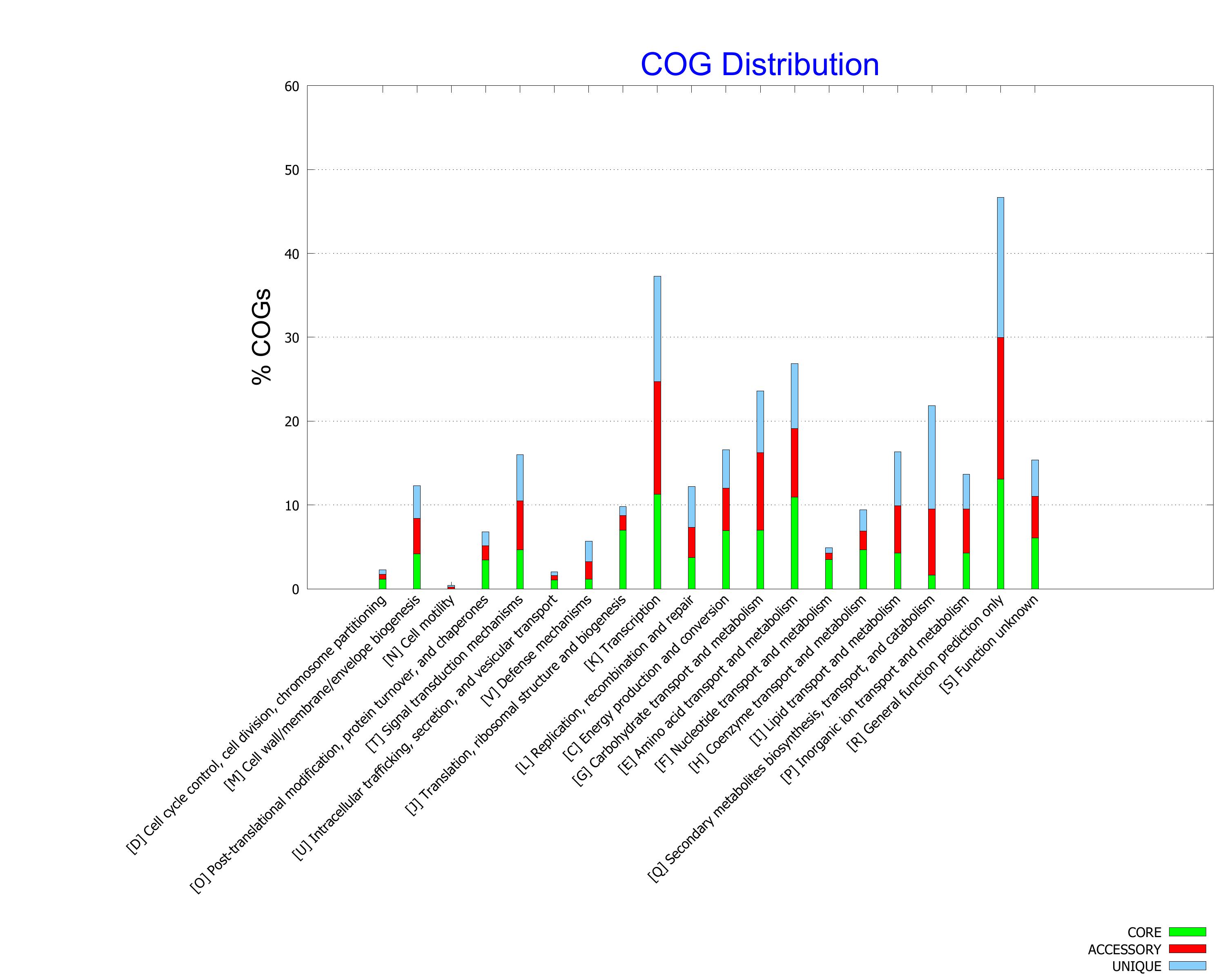

Supplement: Supplementary file 2 [file Data_Sheet_1.ZIP › figures1/COG.jpg]

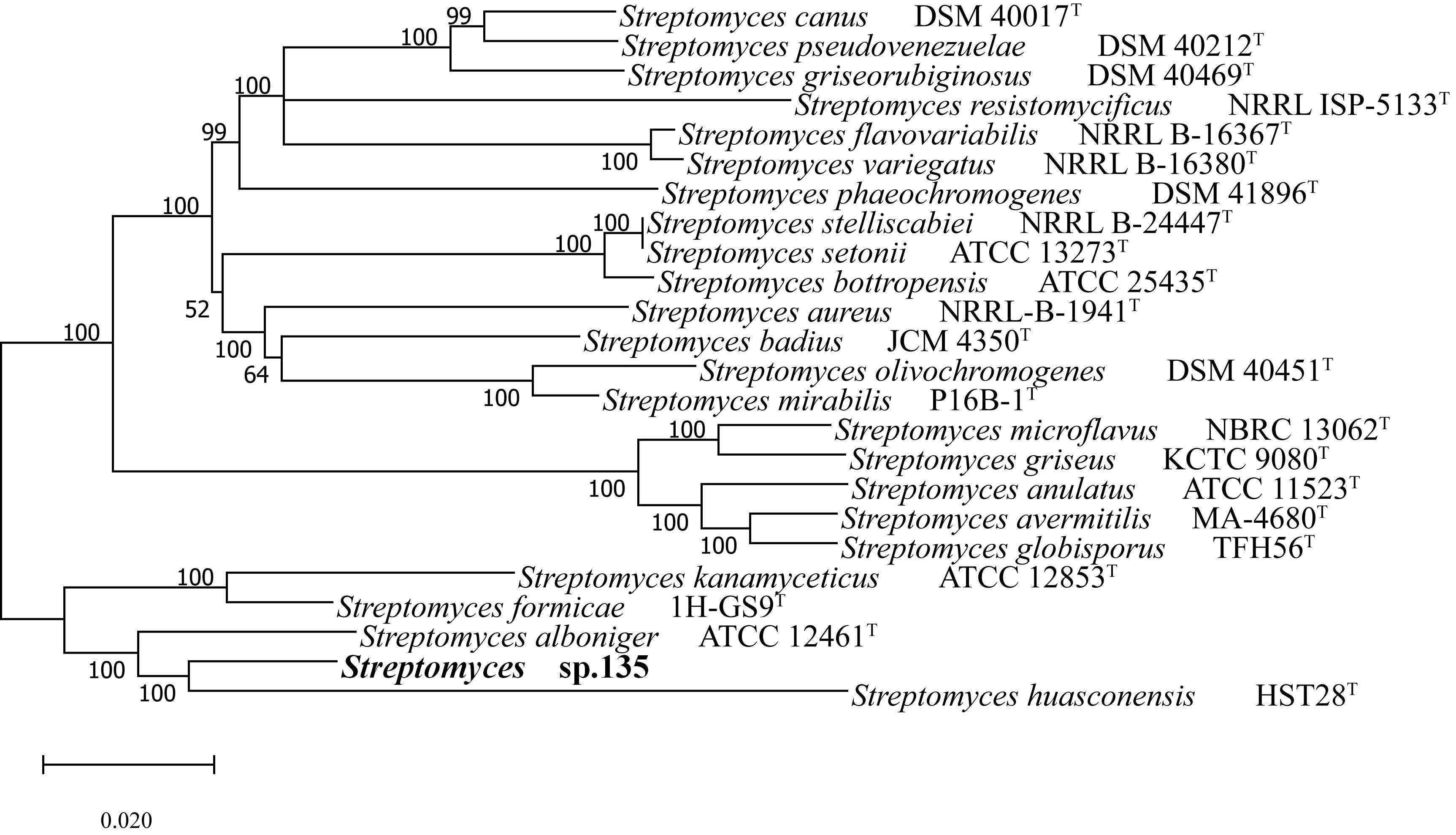

Supplement: Supplementary file 2 [file Data_Sheet_1.ZIP › figures1/core-genome tree.jpg]

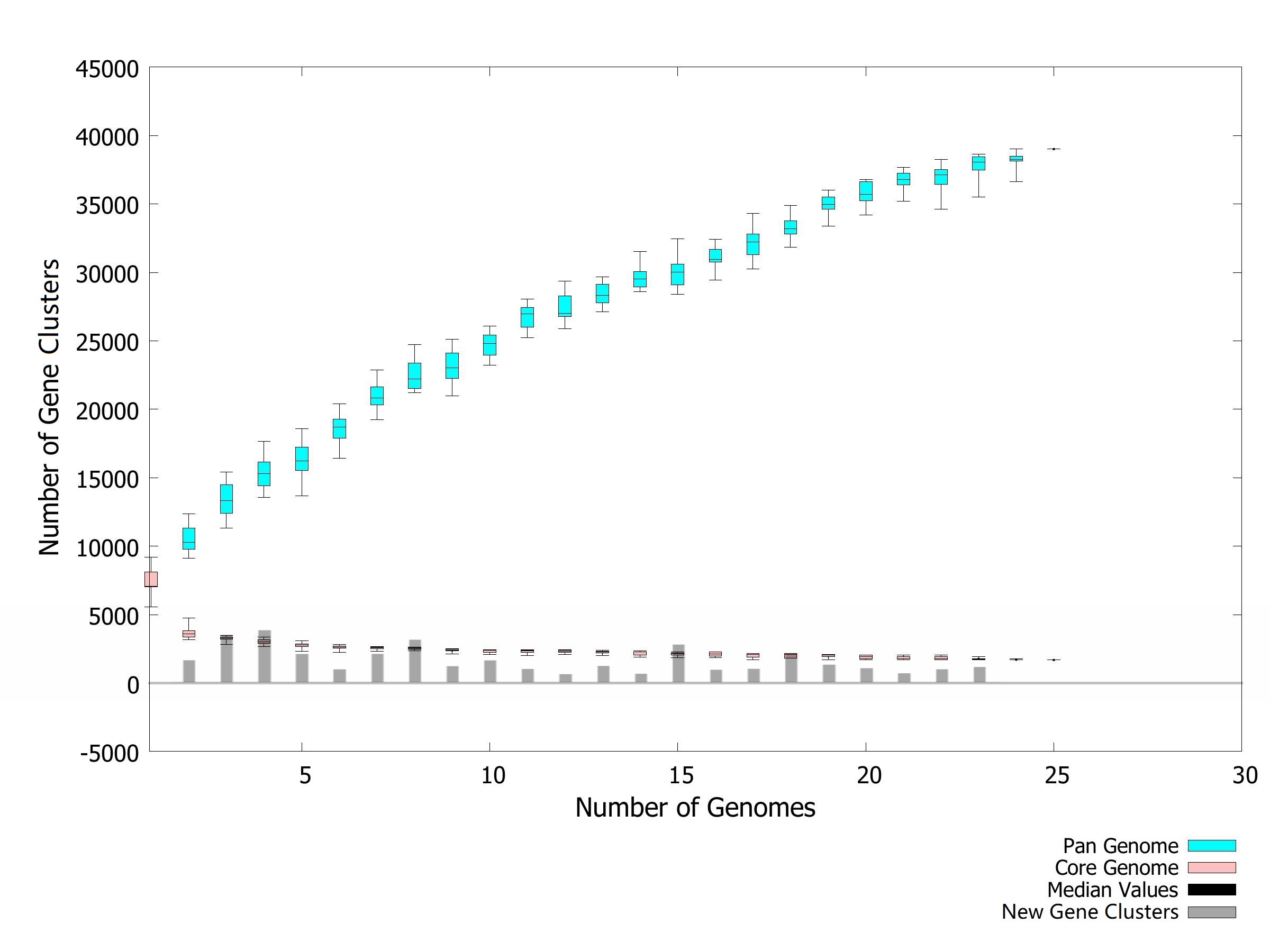

Supplement: Supplementary file 2 [file Data_Sheet_1.ZIP › figures1/core-pan plot.jpg]

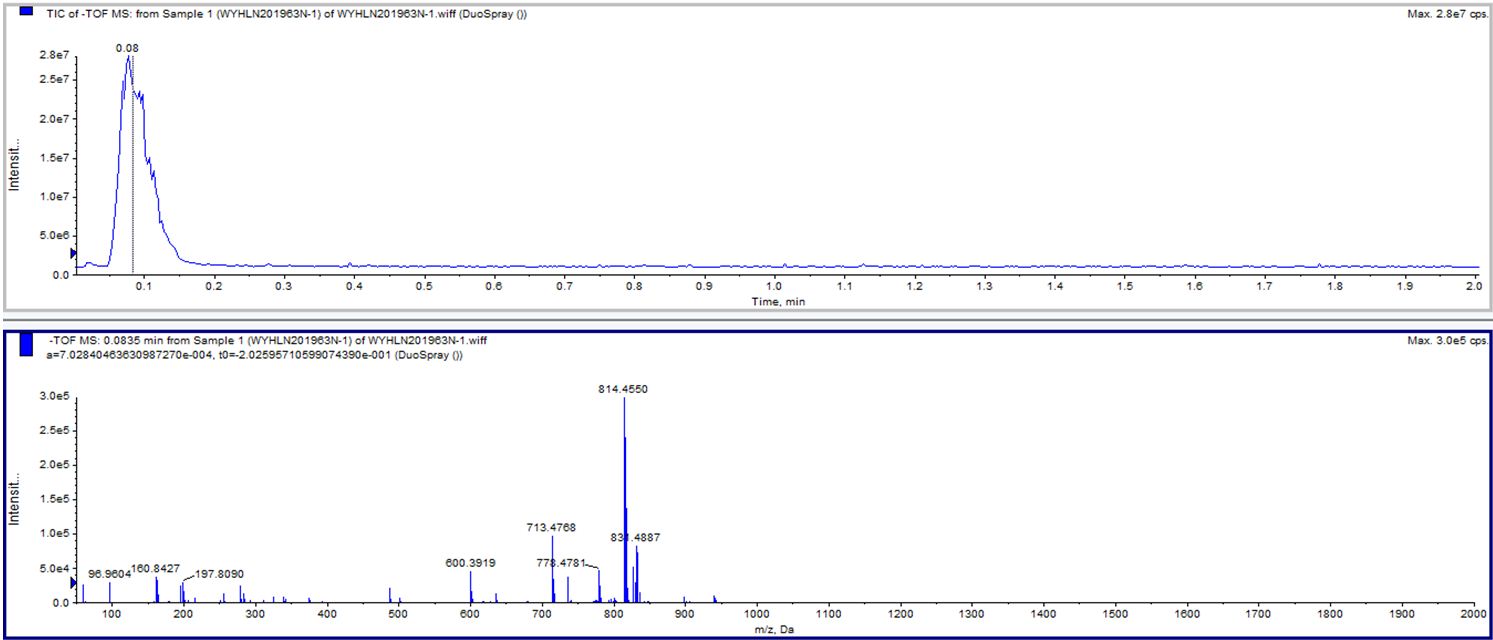

Supplement: Supplementary file 2 [file Data_Sheet_1.ZIP › figures1/HREMS1.JPG]

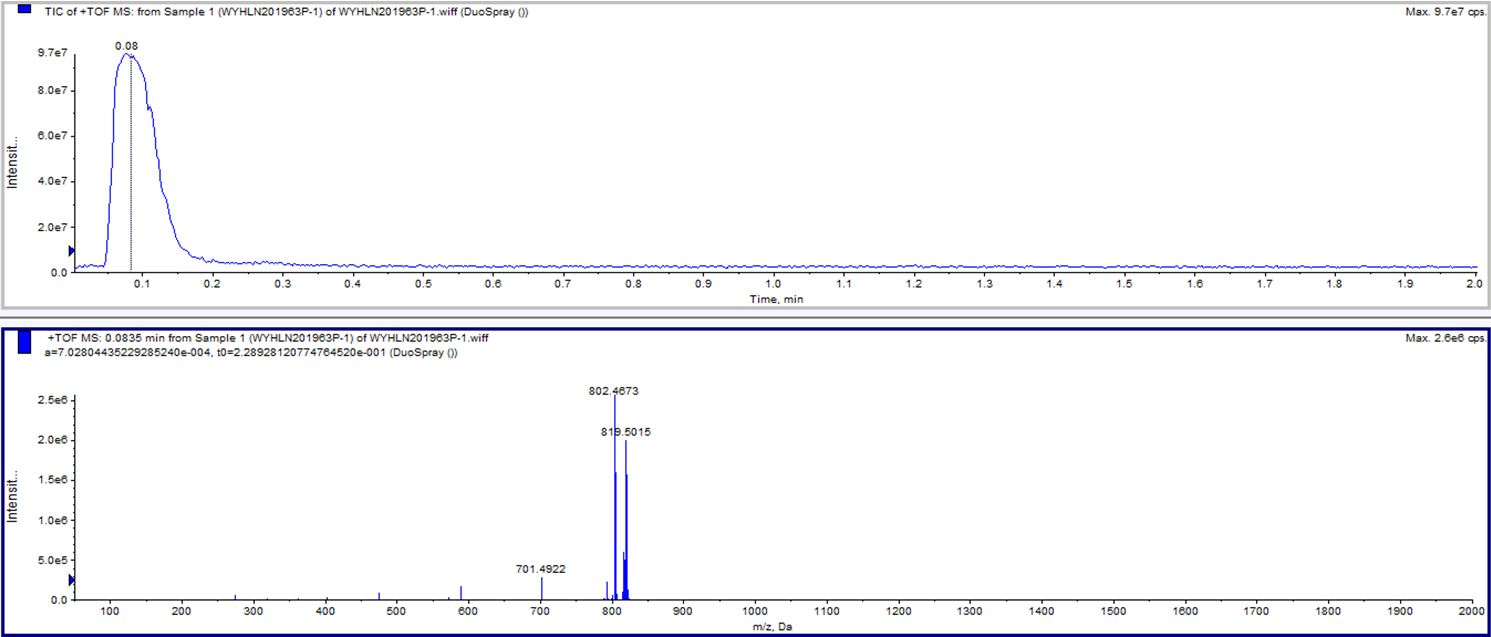

Supplement: Supplementary file 2 [file Data_Sheet_1.ZIP › figures1/HREMS2.JPG]

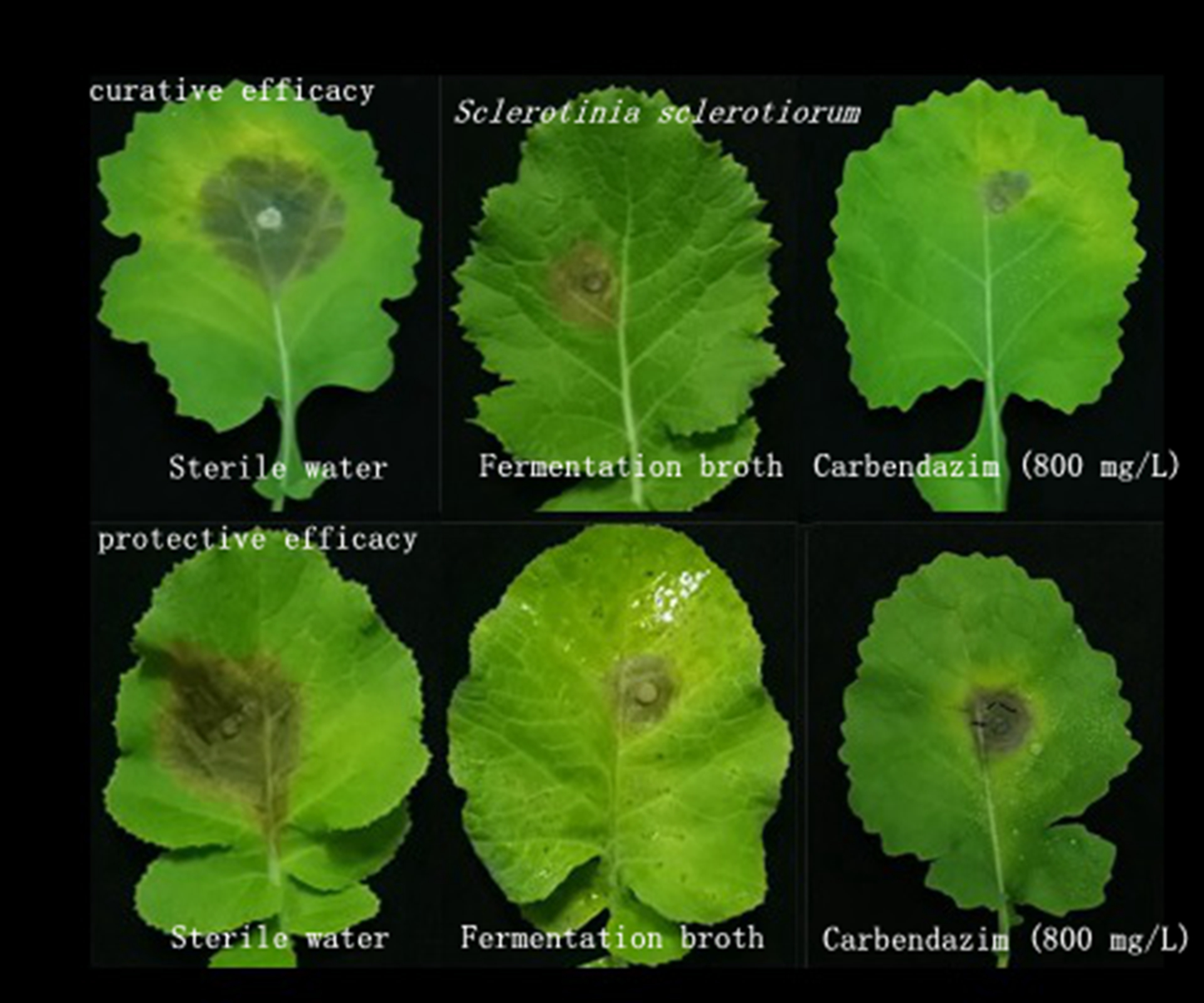

Supplement: Supplementary file 2 [file Data_Sheet_1.ZIP › figures1/rape leaves.jpg]

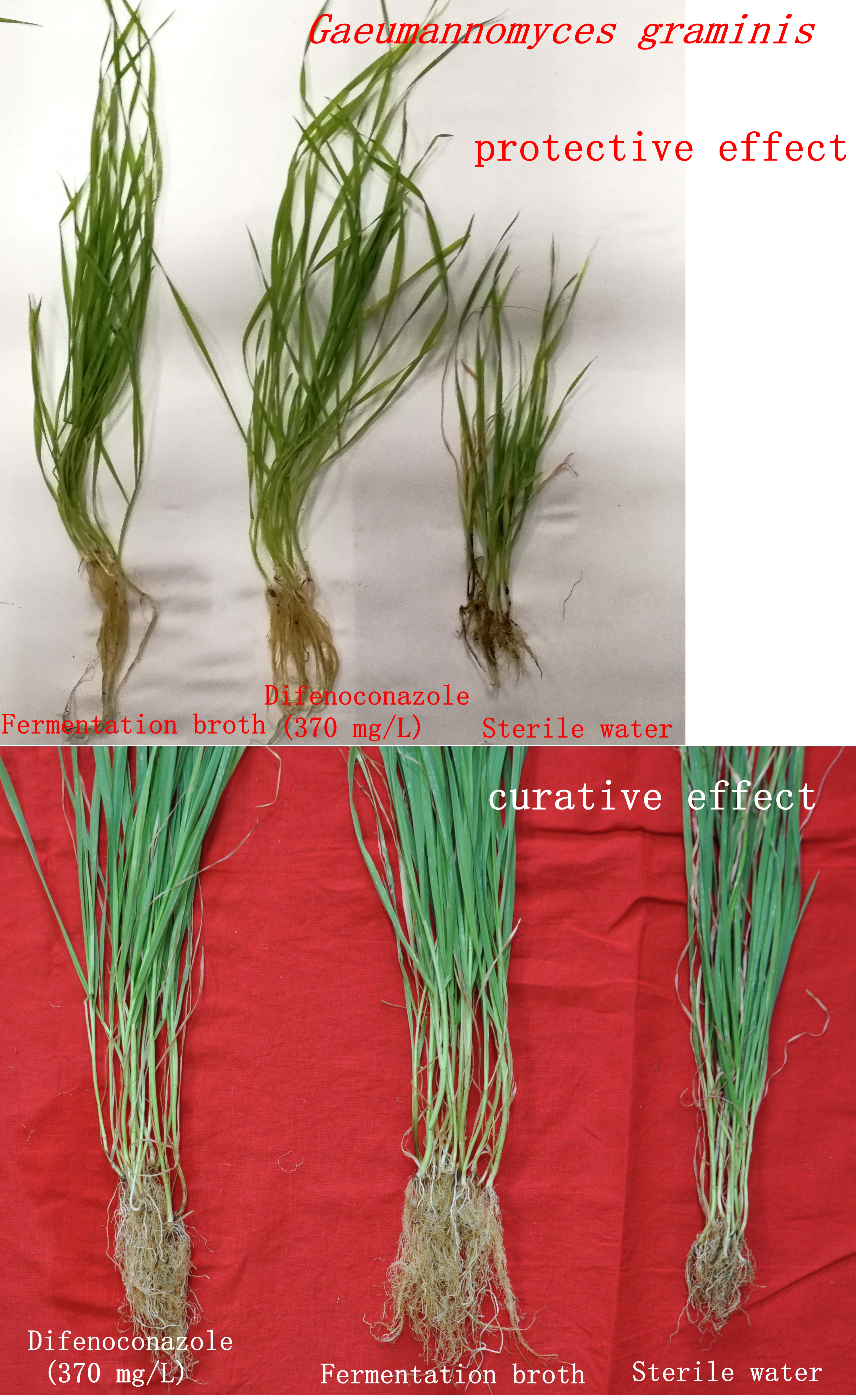

Supplement: Supplementary file 2 [file Data_Sheet_1.ZIP › figures1/wheat.jpg]

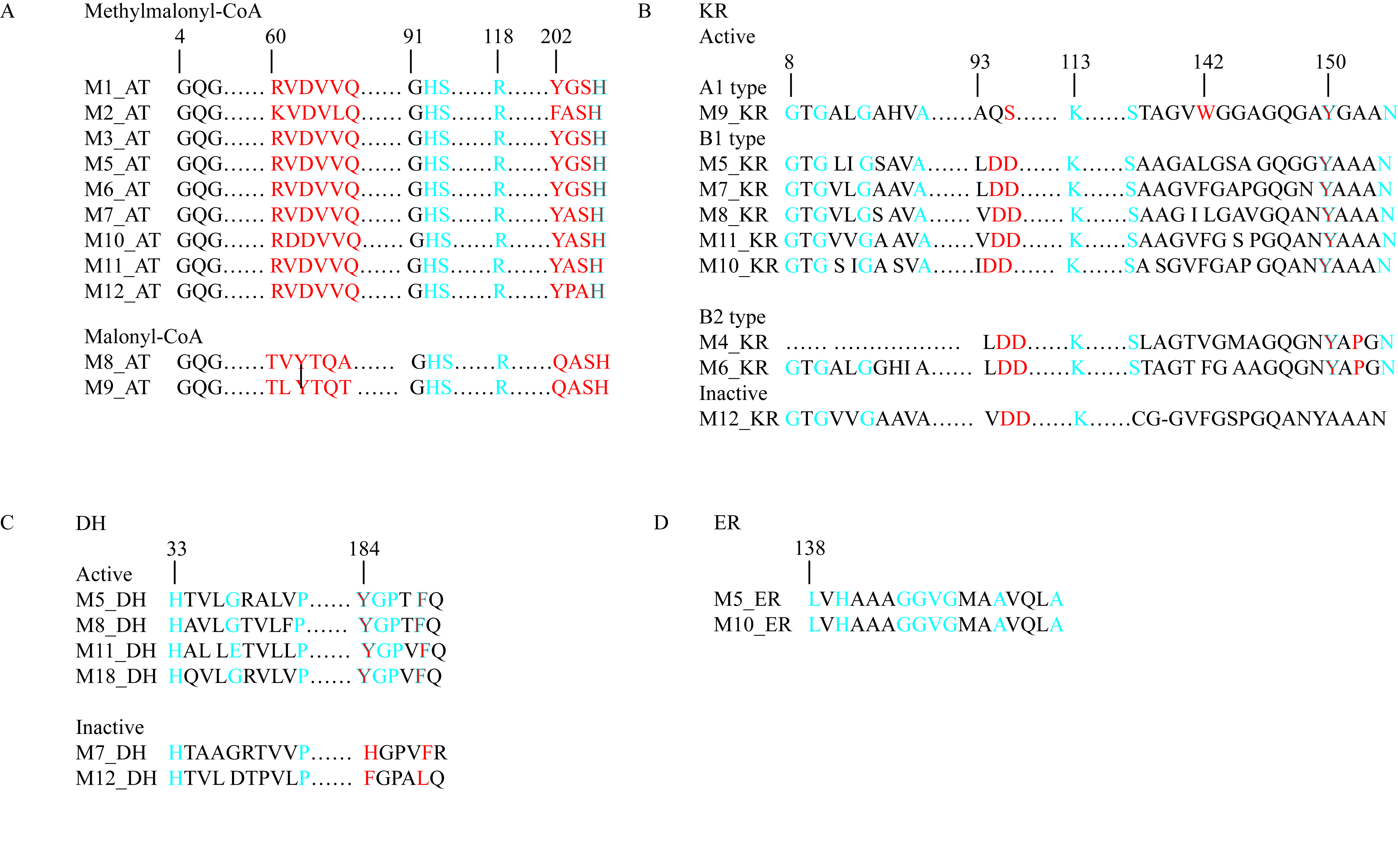

Supplement: Supplementary file 3 [file Data_Sheet_2.ZIP › figures2/Incomplete amino acid sequence alignment of AT, KR, DH, and ER domains in X-14952B biosynthesis.jpg]

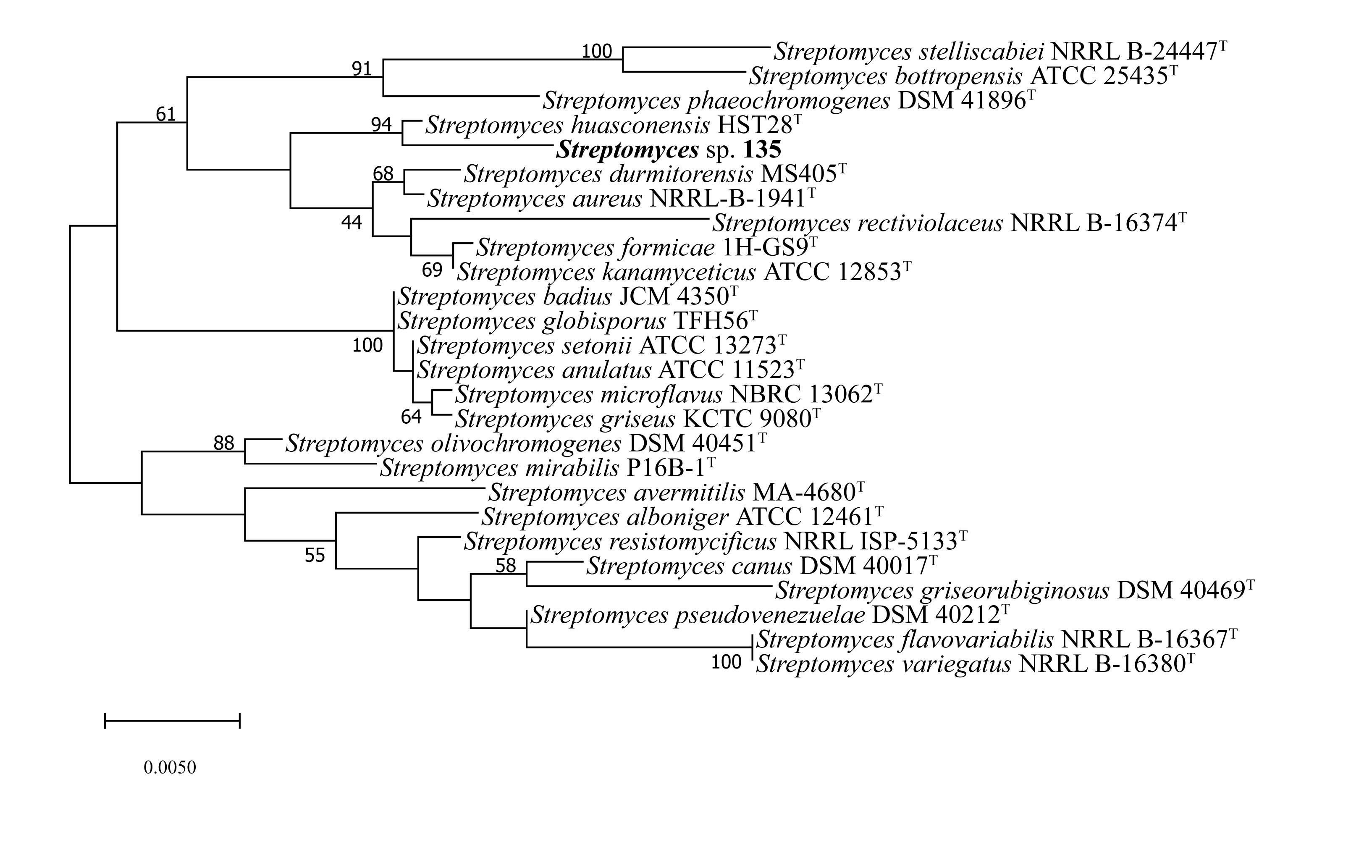

Supplement: Supplementary file 3 [file Data_Sheet_2.ZIP › figures2/MLtree.jpg]

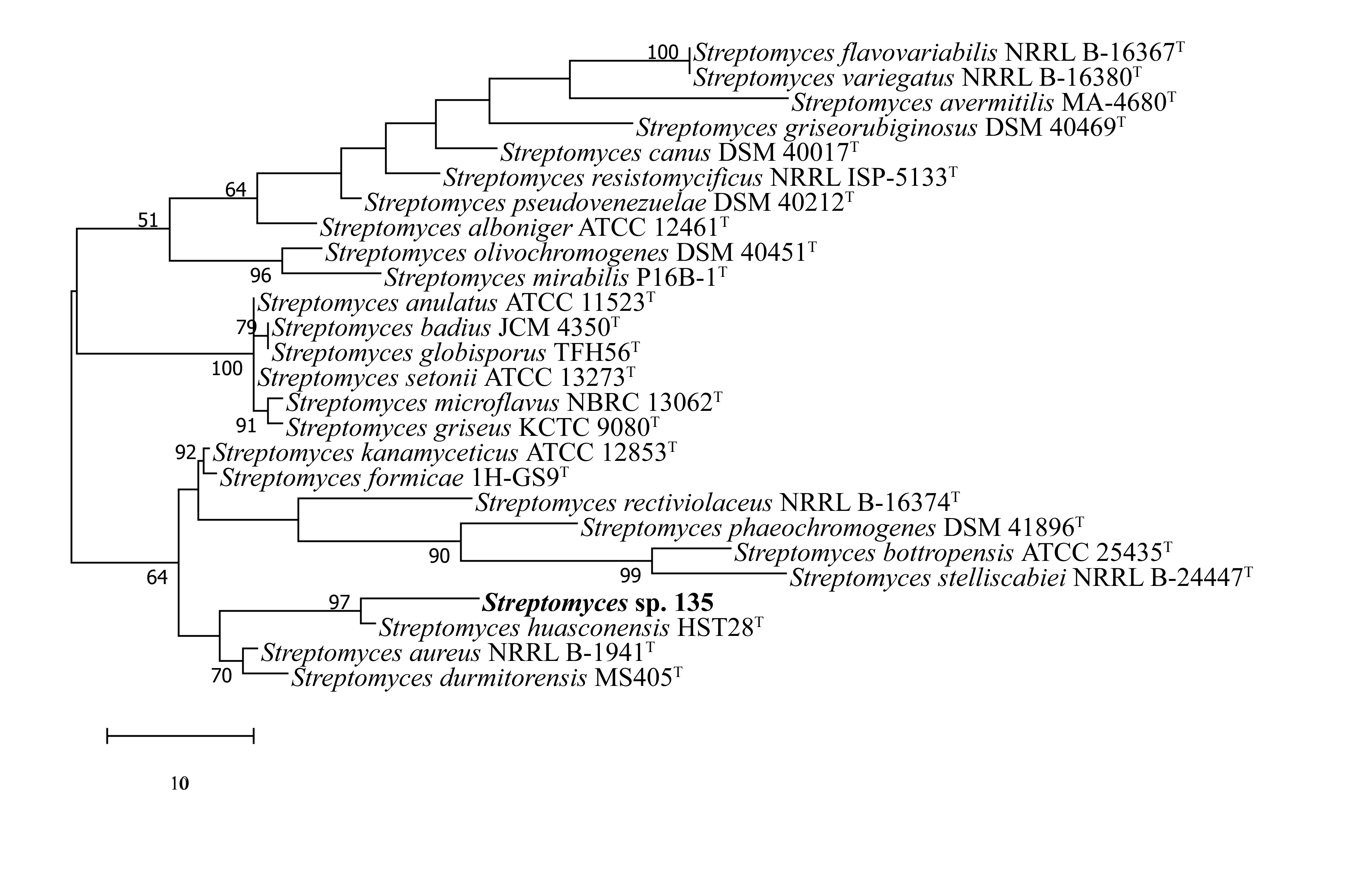

Supplement: Supplementary file 3 [file Data_Sheet_2.ZIP › figures2/MPtree.jpg]

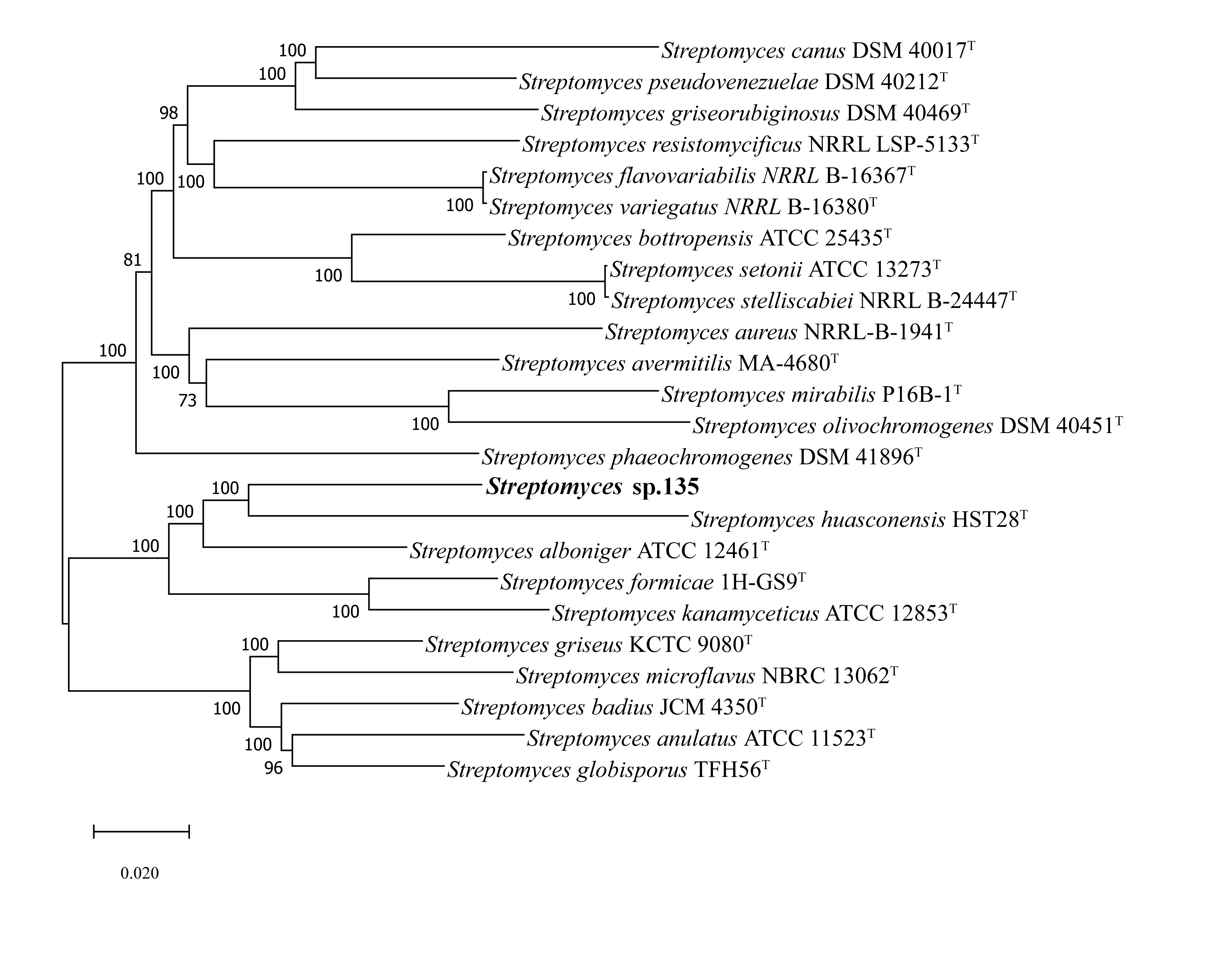

Supplement: Supplementary file 3 [file Data_Sheet_2.ZIP › figures2/pan-genome tree.jpg]

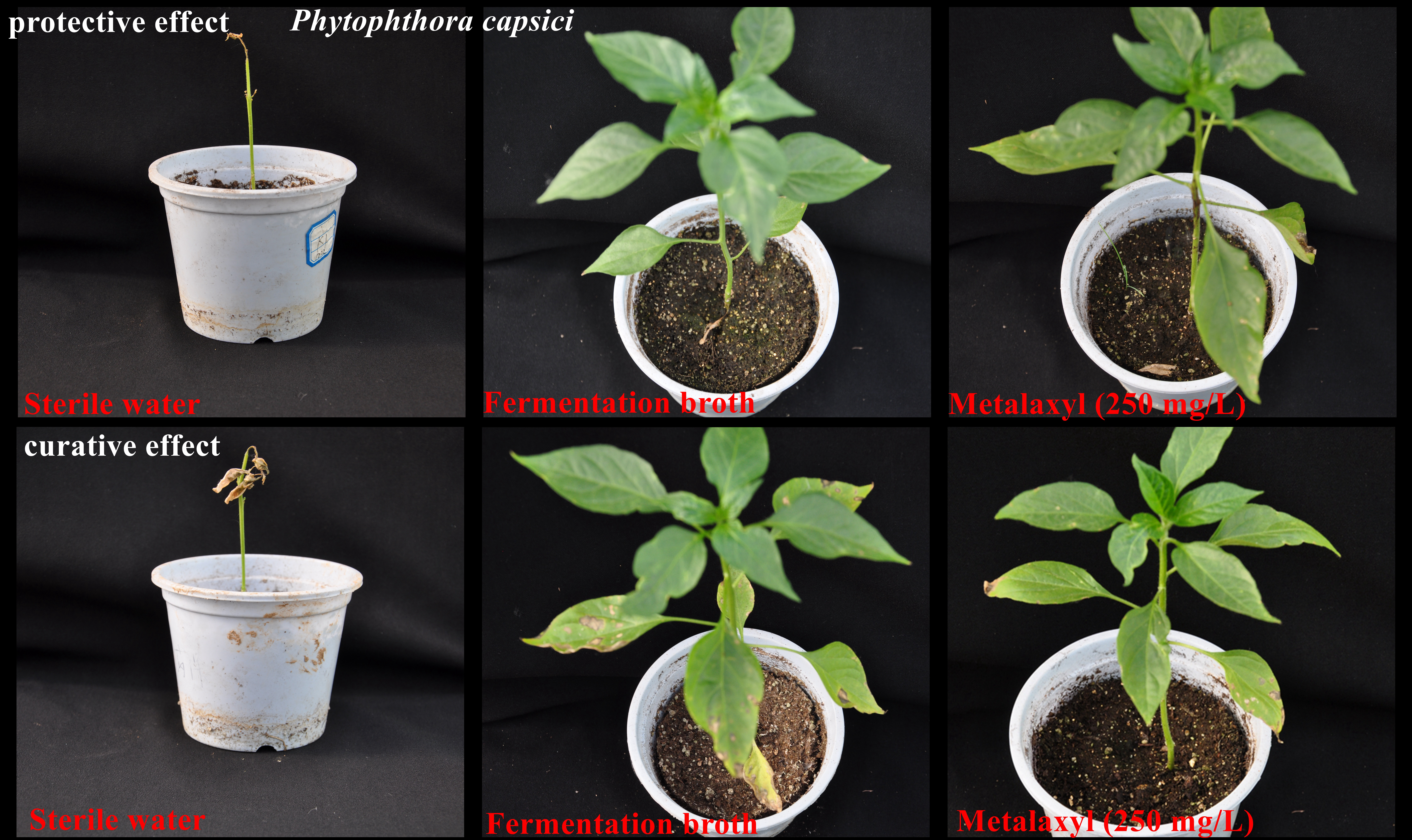

Supplement: Supplementary file 3 [file Data_Sheet_2.ZIP › figures2/pepper.jpg]

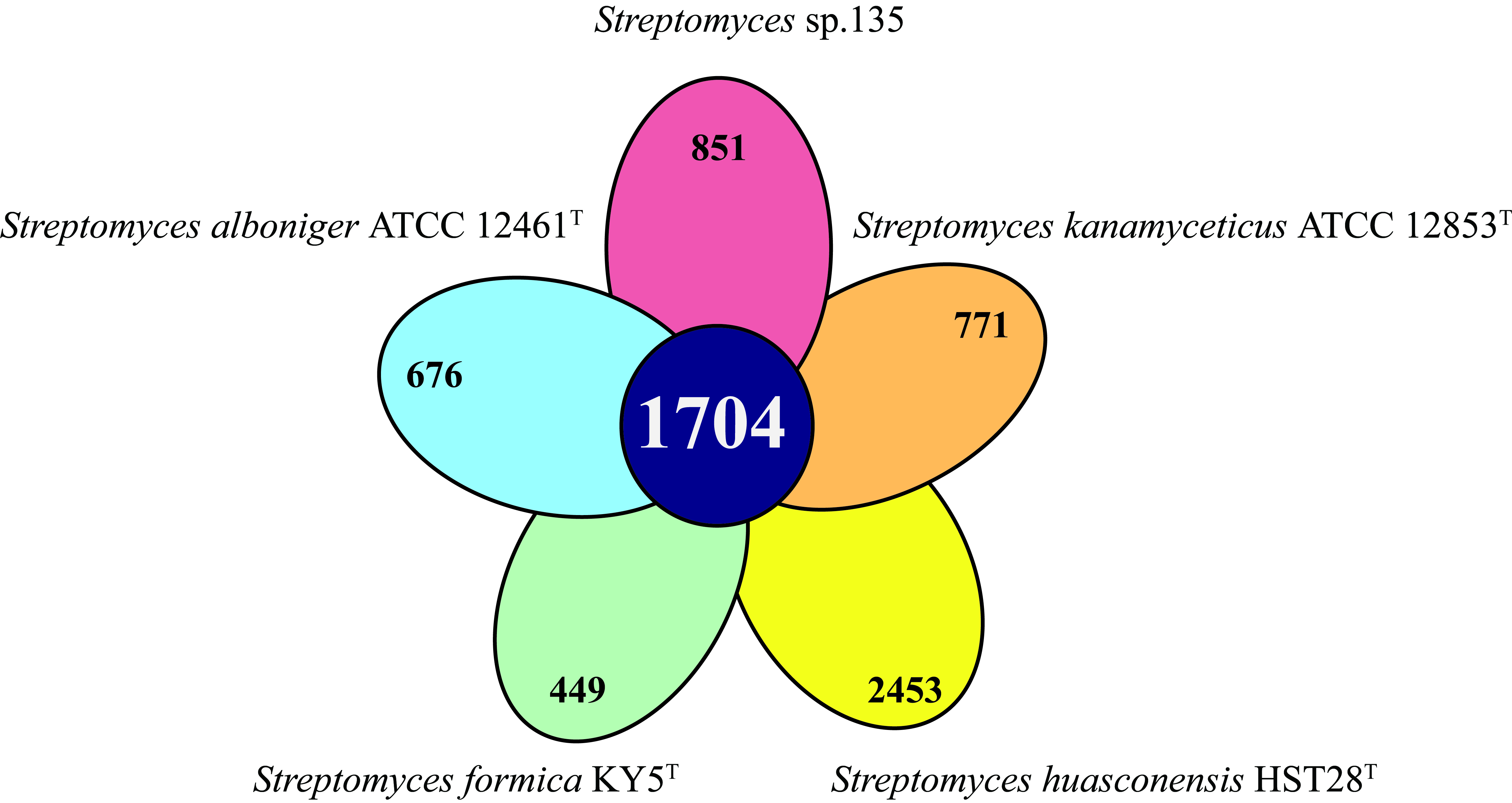

Supplement: Supplementary file 3 [file Data_Sheet_2.ZIP › figures2/pertal diagram.jpg]
